# Supplementary material for: High-fidelity photonic quantum logic gate based on near-optimal Rydberg single-photon source
Source: Nat Commun. 2022 Aug 1;13:4454. doi: 10.1038/s41467-022-32083-9 (PMC9343406; doi:10.1038/s41467-022-32083-9)
Supplement: Supplementary file 1 — Supplementary Information [file 41467_2022_32083_MOESM1_ESM.pdf]

Supplementary Information: High-fidelity photonic quantum logic gate based on near-optimal Rydberg single-photon source

Shuai Shi[1,*], Biao Xu[1,*], Kuan Zhang[1,*], Gen-Sheng Ye[1,*], De-Sheng Xiang[1], Yubao Liu[1], Jingzhi Wang[1], Daiqin Su[1,†], Lin Li[1,‡]
[1]*MOE Key Laboratory of Fundamental Physical Quantities Measurement, Hubei Key Laboratory of Gravitation and Quantum Physics, PGMF, Institute for Quantum Science and Engineering, School of Physics, Huazhong University of Science and Technology, Wuhan 430074, China*
* These authors contributed equally: Shuai Shi, Biao Xu, Kuan Zhang, Gen-Sheng Ye.
[†]e-mail: sudaiqin@hust.edu.cn
[‡]e-mail: li_lin@hust.edu.cn

CONTENTS

I. Supplementary Note 1: Single-photon efficiency and purity 1

II. Supplementary Note 2: Single-Photon indistinguishability 2

III. Supplementary Note 3: High fidelity photon-photon quantum logic gate 6

IV. Supplementary Note 4: Entanglement generation, quantum state tomography and violation of Bell's inequality 12

V. Supplementary Note 5: Potential applications 14

References 16

I. SUPPLEMENTARY NOTE 1: SINGLE-PHOTON EFFICIENCY AND PURITY

In this section, we present the characterization of our single-photon source efficiency and purity.

To characterize the efficiency of our Rydberg single-photon source, we create Rydberg excitation by driving a collective Rabi oscillation between states $|\mathrm{G}\rangle$ and $|\mathrm{R}\rangle$, and measure η_d, the probability of detecting a single photon per experimental cycle. Supplementary Figure 1 displays η_d as a function of the Rabi oscillation driving time t, at the repetition rate of 20 kHz. As a result of Rydberg blockade among N atoms, the two-photon Rabi frequency is collectively enhanced by a factor of $\sqrt{N}$. The effective number of atoms $N \approx 440$ is extracted from a fit to the data in Supplementary Figure 1. With a π-excitation, a peak probability of photoelectric detection $\eta_\mathrm{d} = 0.1035(5)$ is obtained by averaging the number of photon counting events registered by the SPCM during every single-photon readout. Considering the SPCM detection efficiency $\xi_\mathrm{d} = 0.73(1)$ and the overall optical transmission $\xi_\mathrm{t} = 0.47(2)$, which includes the losses of optical elements, AOM diffraction efficiency and fiber coupling efficiency, we estimate the single-photon generation efficiency $\eta = \eta_\mathrm{d}/(\xi_\mathrm{t}\xi_\mathrm{d}) = 0.30(1)$. The single-photon generation rate R is proportional to η and the experimental repetition rate. Higher repetition rate can increase R but also reduce the generation efficiency η due to atomic loss. Single-photon generation rate as high as $R = 5.42(24) \times 10^4\,\mathrm{s}^{-1}$ can be achieved with a repetition rate of 200 kHz. The single-photon generation efficiency is currently limited by the finite matter-light quantum conversion efficiency and by the decoherence during the Rydberg excitation and storage. The matter-light conversion efficiency can in principle be improved by increasing the OD. However, high density could cause severe density-dependent dephasing. Indeed, we have observed a faster dephasing rate with higher atomic density in the current experiment. Ideally, integrating the experiment with a low to medium finesse optical resonator could improve the conversion efficiency while maintaining a low atomic density and longer coherence times.

The high purity of our single photons is guaranteed by the strong van der Waals interactions between the high-lying Rydberg atoms which effectively suppress multiple excitations within the blockade radius r_b. For the Rydberg state $|90\mathrm{S}_{1/2}, J = 1/2, m_\mathrm{J} = 1/2\rangle$ used in the experiment, the blockade radius is $r_\mathrm{b} \approx 15\,\mu\mathrm{m}$. The waists of the

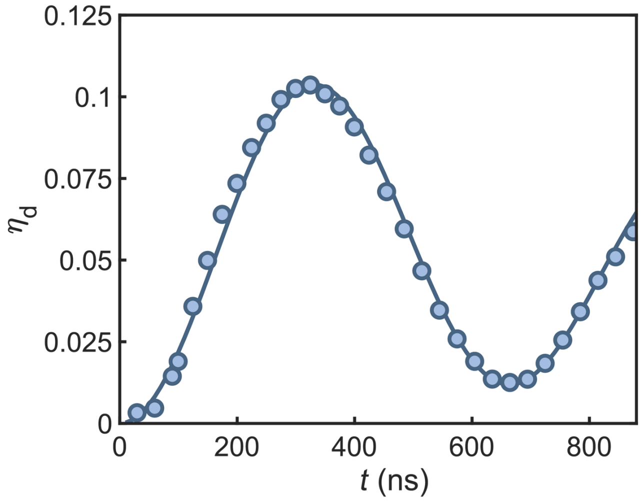

Supplementary Figure 1. Characterization of the Rydberg single-photon source efficiency. Probability of detecting a single photon per experimental trial η_d is shown as a function of Rydberg excitation time t. The solid curve is a fit based on many-body Rabi oscillation.

Rydberg excitation and dipole trap beams are chosen such that the entire excitation region is within the Rydberg blockade radius and only single excitation is allowed. To characterize the purity of the generated single photons, a Hanbury Brown-Twiss (HBT) experiment is performed to measure the second-order intensity correlation function at zero delay $g^{(2)}(0)$ of the single photon. The read-out laser converts the Rydberg single excitation into a high-quality single photon, which is coupled into a 50:50 fiber beam splitter and detected by a pair of SPCMs with lower dark-count rates. The quantum statistics of the single-photon field is then obtained by analyzing the distribution of the photoelectric detection events

$$g^{(2)}(\tau) = \frac{p_{12}(\tau)}{p_1(0)p_2(\tau)}, \tag{1}$$

where p_1 and p_2 are the photoelectric detection probabilities of each SPCM, and $p_{12}(\tau)$ is the probability of coincidences between two SPCMs with delay τ. The value of second-order intensity correlation function at zero delay, $g^{(2)}(0)$, is a direct measure of single-photon purity, since $g^{(2)}(0)$ is zero for an ideal single photon. Besides residual multi-photon events, background detection events also contribute to the non-zero $g^{(2)}(0)$ measured in the HBT experiment. In order to suppress the background detection events to SPCMs dark-count level, we employ a pair of PBSs for polarization filtering, a 12.5 nm bandwidth filter and a Fabry-Perot etalon for frequency filtering, and a single-mode fiber for spatial filtering, which bring extra loss of about 0.62(3). To further increase the signal-to-background ratio of photoelectric detection, a 200 ns detection gating window, which contains 94 % of photons around the center, is applied to reduce the dark counts per trial. The measured second-order intensity correlation function at zero delay is $g^{(2)}(0) = 7.5(6) \times 10^{-4}$, indicating near-unity single-photon purity. Given that the probability of coincidences between two background detection events is negligible, the contribution of background detection events to $g^{(2)}(0)$ is given by $p_1^{\mathrm{B}}/p_1 + p_2^{\mathrm{B}}/p_2 = 2.7(2) \times 10^{-4}$, where p_1^{B} and p_2^{B} are the probabilities of background detection events for the SPCMs. Considering the contribution from background detection events, we extract the background-corrected second-order intensity correlation function $g_{\mathrm{cor}}^{(2)}(0) = 4.8(6) \times 10^{-4}$.

II. SUPPLEMENTARY NOTE 2: SINGLE-PHOTON INDISTINGUISHABILITY

In this section, we present detailed description and analysis of Hong–Ou–Mandel (HOM) experiment to investigate the two-photon quantum interference and characterize the photon indistinguishability.

The two photons used in the HOM experiment are sequentially generated from the Rydberg atoms with an interval of 5 µs, and temporally matched by a polarization switching electro-optic modulator (EOM) and a 1 km delay fiber (See Supplementary Figure 2). Good temporal mode-matching between the two-photon wave packets is a prerequisite

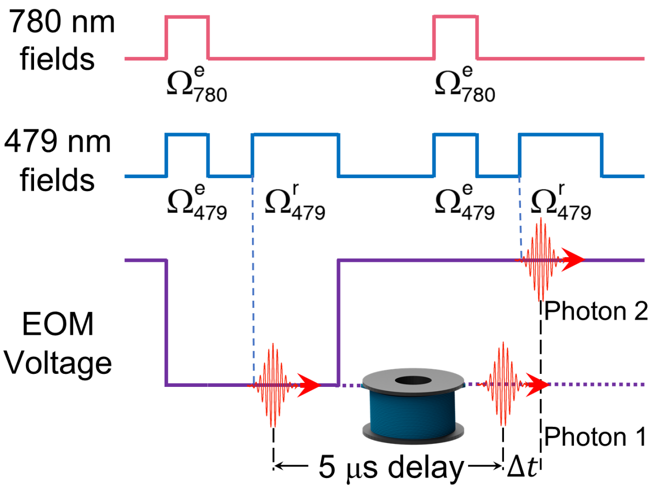

Supplementary Figure 2. Timing sequence for HOM experiment. The single-photon generation protocol is executed twice in every experimental cycle with a delay of 5 µs. To ensure the temporal wave-packet matching of two photons, a polarization switching EOM and a 1 km fiber are used to delay the first photon by 5 µs. The temporal mismatch Δt between two photons is varied to measure the HOM dip in Fig. 2b.

for high interference visibility. We characterize the temporal mode-matching with a wave-packet overlap function

$$\left(\int_0^\infty |\psi_1^*(t)\psi_2(t)|dt\right)^2 = \frac{\left(\int_0^\infty \sqrt{I_1(t)I_2(t)}dt\right)^2}{\int_0^\infty I_1(t)dt \int_0^\infty I_2(t)dt}, \tag{2}$$

where $\psi_1(t)$ and $\psi_2(t)$ are the temporal mode functions of two photons, and $I_1(t)$ and $I_2(t)$ are their intensity distributions over time. We measure $I_1(t)$ and $I_2(t)$, and obtain a wave-packet overlap function with the value of 99.946(8) % for 200 ns detection window and 99.970(11) % for 80 ns detection window. Moreover, we analyze the wave-packet overlap function with a temporal mismatch Δt, $(\int \sqrt{I_1(t)I_2(t+\Delta t)}dt)^2$, and find its dependence on Δt can be well approximated with a Gaussian function, which is also used to fit the data in Fig. 2b in the main text.

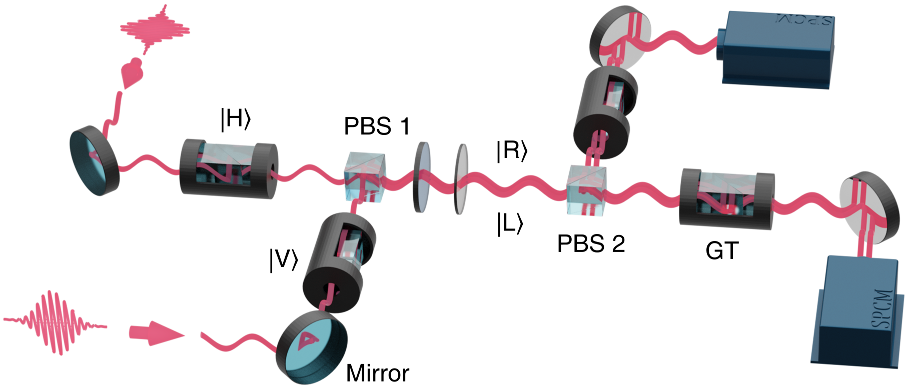

Supplementary Figure 3. Experimental setup of the Hong–Ou–Mandel interference. Two photons in orthogonally polarized states |H⟩ and |V⟩ are combined on the PBS 1 and transformed to circularly polarized states |L⟩ and |R⟩ before PBS 2. Four Glan-Taylor polarizers are employed to ensure high polarization extinction ratios. A pair of SPCMs are used to measure the two-photon interference visibility.

Having two single photons with an excellent temporal overlap at hand, we proceed to the implementation of the HOM experiment, as shown in Supplementary Figure 3. In order to fine tune the beam splitting ratio to be precisely 50:50, we build a HOM setup in which two single photons with orthogonal circular polarizations interfere at a PBS. Using Glan-Taylor polarizers, two single photons are prepared in linearly polarized states |H⟩ and |V⟩ with high

polarization extinction ratios and then combined at PBS 1. After being rotated to circularly polarized states $|\mathrm{R}\rangle$ and $|\mathrm{L}\rangle$, two single photons interfere at PBS 2 and are detected by a pair of SPCMs. Two more Glan-Taylor polarizers and single-mode fibers (not shown) are used before the SPCMs to suppress the polarization crosstalk and provide spatial filtering, respectively.

The HOM interferometer establishes an input-output relation of

$$\begin{pmatrix} \hat{t}_{\mathrm{H}} \\ \hat{r}_{\mathrm{V}} \end{pmatrix} = \frac{1}{\sqrt{2}} \begin{pmatrix} 1 & i \\ i & 1 \end{pmatrix} \begin{pmatrix} \hat{a}_{\mathrm{L}} \\ \hat{a}_{\mathrm{R}} \end{pmatrix}, \tag{3}$$

where $\hat{a}_{\mathrm{L}}$ ($\hat{a}_{\mathrm{R}}$) represents the left (right) circularly polarized input mode, and $\hat{t}_{\mathrm{H}}$ ($\hat{r}_{\mathrm{V}}$) represents the transmitted (reflected) output mode. When two single photons with left and right circular polarizations arrive at PBS 2 simultaneously, the HOM interference results in a transformation of $\hat{a}_{\mathrm{L}}^{\dagger}\hat{a}_{\mathrm{R}}^{\dagger} \to -i(\hat{t}_{\mathrm{H}}^{\dagger}\hat{t}_{\mathrm{H}}^{\dagger} + \hat{r}_{\mathrm{V}}^{\dagger}\hat{r}_{\mathrm{V}}^{\dagger})/2$. Ideally, two single photons should appear together at the same output port, and the probability of coincidence between different ports should be zero. However, background detection events, single-photon impurity, photon indistinguishability and imperfections of optical components lead to non-zero coincidences, and thus reduce the HOM interference visibility.

As mentioned in the main text, we find that the reduction of indistinguishability from the unit at zero delay is mainly due to the imperfect interference from photon components in the rising and falling edges of the single-photon temporal profile. Here, we study this effect by performing the HOM experiment with different photon detection windows T_d, as shown in Supplementary Figure 4. Our single photon has a temporal profile with a full width at half maximum (FWHM) of about 100 ns. As the detection window decreases from 200 ns to 80 ns around the center, the HOM visibility (indistinguishability) at zero delay is improved from 99.43(9) % (99.55(9) %) to 99.87(8) % (99.94(8) %), although their wave-packet overlap function has a negligible difference. These results suggest the possibility of the phase chirps in the rising and falling edges of the single-photon temporal profile. With the 80 ns detection window, 62 % of the photons in the single-photon wave packet are detected and the $g^{(2)}(0)$ is improved from $7.5(6) \times 10^{-4}$ (200 ns window) to $4.5(6) \times 10^{-4}$ due to a better signal to background ratio. From a single-photon application point of view, it is ideal to apply a proper detection window that: (1). gives high signal-to-background ratio, photon purity and interference visibility; (2). contains a large percentage of the photons. Therefore, we use the 200 ns detection window for most of the single-photon characterization measurements and employ the 80 ns detection window for the implementation of the CNOT gate.

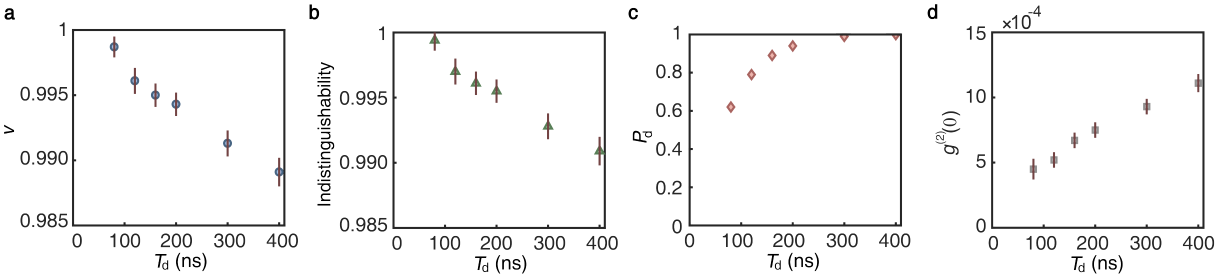

Supplementary Figure 4. Single-photon properties with different detection windows. a, The visibility of HOM interference at zero temporal delay $V(0)$ as a function of detection window T_d; **b,** The photon indistinguishability as a function of detection window T_d; **c,** The percentage of the single-photon wave-packet P_d that falls into the detection window T_d; **d,** The Second-order intensity correlation function at zero delay $g^{(2)}(0)$ as a function of detection window T_d. The error bars in **(a)**, **(b)** and **(d)** represent 1σ standard deviation.

In principle, the imperfect interference in the edges can be compensated, so a larger detection window and higher single-photon detection efficiency can be achieved. While the photon counting measurement employed here only gives the amplitude distribution of the single-photon wave packet, a homodyne detection setup could provide the complex temporal profile of the single photon, including the phase information. We plan to implement such a homodyne detection protocol to extract phase distribution inside the single-photon profile to investigate the possible phase chirps. The phase chirps can in principle be compensated by optimally controlling the phase of the 479 nm read-out field. However, it goes beyond the scope of the current work.

Now we proceed to the analysis of HOM interference visibility with 80 ns detection window. The visibility of HOM quantum interference is given by $V(t) = 1 - C(t)/C(\infty)$, where $C(t)$ is the measured probability of two-photon coincidence with a temporal delay t, and $C(\infty)$ is the expected probability of coincidence if two photons are completely distinguishable. We evaluate $C(\infty)$ by inputting the left and right circularly polarized single photons separately. From the photoelectric detection probabilities of the transmitted (reflected) photons $p_{\mathrm{t}}^{\mathrm{L}} = 0.006275(20)$

($p_{\rm r}^{\rm L}=0.006576(21)$) and $p_{\rm t}^{\rm R}=0.002281(12)$ ($p_{\rm r}^{\rm R}=0.002406(13)$), we have $C(\infty)=p_{\rm t}^{\rm L}p_{\rm r}^{\rm R}+p_{\rm r}^{\rm L}p_{\rm t}^{\rm R}=3.010(13)\times10^{-5}$. At zero temporal delay, we obtain a minimal probability of coincidences $C(0)=4.0(23)\times10^{-8}$ and a maximal HOM interference visibility $V(0)=99.87(8)\,\%$. Next, we analyze the major mechanisms that cause reduction of the HOM interference visibility, and extract the indistinguishability of our Rydberg single-photon source.

Visibility reduction due to background detection events. The background detection events, which mainly consist of dark counts from SPCMs, contribute to the undesired coincidences in the HOM experiment. Similar to the analysis in Section I, the probability of detecting coincidence caused by background per experimental trial is $p_{\rm t}p_{\rm r}^{\rm B}+p_{\rm r}p_{\rm t}^{\rm B}$, where $p_{\rm t}$ ($p_{\rm r}$) represents the photoelectric detection probability of the transmitted (reflected) photons and $p_{\rm t}^{\rm B}$ ($p_{\rm r}^{\rm B}$) is the probability of background detection events from the SPCM in the transmitted (reflected) output port. These undesired coincidences decrease the HOM interference visibility from unity by

$$\Delta V_{\rm bg}\approx\frac{p_{\rm t}p_{\rm r}^{\rm B}+p_{\rm r}p_{\rm t}^{\rm B}}{p_{\rm t}^{\rm L}p_{\rm r}^{\rm R}+p_{\rm r}^{\rm L}p_{\rm t}^{\rm R}}=0.034(8)\,\%, \tag{4}$$

where $p_{\rm t}=0.008280(11)$, $p_{\rm r}=0.008671(11)$, $p_{\rm t}^{\rm B}=4.7(18)\times10^{-7}$ and $p_{\rm r}^{\rm B}=7.3(22)\times10^{-7}$.

Visibility reduction due to single-photon impurity. The multi-photon components of our single-photon source, characterized by $g^{(2)}(0)$, also cause a reduction of interference visibility. The multi-photon components increase the probability of coincidence by

$$\langle\hat r_{\rm V}^\dagger\hat t_{\rm H}^\dagger\hat t_{\rm H}\hat r_{\rm V}\rangle=\frac{\langle\hat a_{\rm L}^\dagger\hat a_{\rm L}^\dagger\hat a_{\rm L}\hat a_{\rm L}\rangle+\langle\hat a_{\rm R}^\dagger\hat a_{\rm R}^\dagger\hat a_{\rm R}\hat a_{\rm R}\rangle+\langle\hat a_{\rm L}^\dagger\hat a_{\rm L}^\dagger\hat a_{\rm R}\hat a_{\rm R}+\hat a_{\rm R}^\dagger\hat a_{\rm R}^\dagger\hat a_{\rm L}\hat a_{\rm L}\rangle}{4}, \tag{5}$$

where the third expectation value is zero since the phases of two input modes $\hat a_{\rm L}$ and $\hat a_{\rm R}$ are unrelated. The reduction of interference visibility due to multi-photon components is then given by

$$\Delta V_{\rm impurity}=\frac{\langle\hat a_{\rm L}^\dagger\hat a_{\rm L}^\dagger\hat a_{\rm L}\hat a_{\rm L}\rangle+\langle\hat a_{\rm R}^\dagger\hat a_{\rm R}^\dagger\hat a_{\rm R}\hat a_{\rm R}\rangle}{2\,\langle\hat a_{\rm L}^\dagger\hat a_{\rm L}\rangle\,\langle\hat a_{\rm R}^\dagger\hat a_{\rm R}\rangle}=\frac{\eta_{\rm L}}{2\eta_{\rm R}}g_{\rm L}^{(2)}(0)+\frac{\eta_{\rm R}}{2\eta_{\rm L}}g_{\rm R}^{(2)}(0)=0.036(10)\,\%. \tag{6}$$

Here, $\eta_{\rm L}$ ($\eta_{\rm R}$) is the probability of having a photon in the input mode $\hat a_{\rm L}$ ($\hat a_{\rm R}$), and the intensity ratio between them is $\eta_{\rm L}/\eta_{\rm R}=2.045(3)$. Since the two single photons used in the HOM experiment are generated from the same source, $g_{\rm L}^{(2)}(0)$ is identical to $g_{\rm R}^{(2)}(0)$, which is measured in Section I with the value of $2.9(8)\times10^{-4}$.

Visibility reduction due to imperfect optical elements. The imperfections of optical elements in the HOM experiment also contribute to the measured coincidences. The imperfections of half-wave plates (HWP) and quarter-wave plates (QWP) induce a deviation of the interferometer beam splitting ratio from 50:50, characterized by $\epsilon_{\rm q}$, and lead to a modified input-output relation

$$\begin{pmatrix}\hat t_{\rm H}\\ \hat r_{\rm V}\end{pmatrix}=\begin{pmatrix}\sqrt{\frac{1+\epsilon_{\rm q}}{2}} & i\sqrt{\frac{1-\epsilon_{\rm q}}{2}}\\ i\sqrt{\frac{1-\epsilon_{\rm q}}{2}} & \sqrt{\frac{1+\epsilon_{\rm q}}{2}}\end{pmatrix}\begin{pmatrix}\hat a_{\rm L}\\ \hat a_{\rm R}\end{pmatrix}. \tag{7}$$

Additionally, the finite extinction ratios of transmission and reflection at PBS 2, denoted by $1:\epsilon_{\rm t}^2$ and $1:\epsilon_{\rm r}^2$, result in a polarization crosstalk between two output modes. The above-mentioned imperfections reduce the HOM interference visibility by

$$\Delta V_{\rm optics}\approx2\frac{\langle\hat r_{\rm V}^\dagger\hat t_{\rm H}^\dagger\hat t_{\rm H}\hat r_{\rm V}\rangle+\epsilon_{\rm r}^2\,\langle\hat t_{\rm H}^\dagger\hat t_{\rm H}^\dagger\hat t_{\rm H}\hat t_{\rm H}\rangle+\epsilon_{\rm t}^2\,\langle\hat r_{\rm V}^\dagger\hat r_{\rm V}^\dagger\hat r_{\rm V}\hat r_{\rm V}\rangle}{\langle\hat a_{\rm L}^\dagger\hat a_{\rm L}\rangle\,\langle\hat a_{\rm R}^\dagger\hat a_{\rm R}\rangle}=2(\epsilon_{\rm q}^2+\epsilon_{\rm r}^2+\epsilon_{\rm t}^2)=0.005(7)\,\%, \tag{8}$$

where $\epsilon_{\rm q}=0.0049(37)$, $\epsilon_{\rm r}=0.001186(2)$ and $\epsilon_{\rm t}=0.000730(1)$ are measured with coherent laser light.

Photon indistinguishability. In order to characterize the indistinguishability of two input photons, we introduce an additional right circularly polarized mode $\hat a_{\rm R}'$, which does not interfere with $\hat a_{\rm L}$, and rewrite the input mode $\hat a_{\rm R}$ as $\sqrt{1-\epsilon_{\rm d}}\hat a_{\rm R}+\sqrt{\epsilon_{\rm d}}\hat a_{\rm R}'$, where $\epsilon_{\rm d}$ quantifies the two-photon distinguishability. The HOM intereference visibility reduction due to the distinguishable input mode $\hat a_{\rm R}'$ is given by

$$\Delta V_{\rm d}\approx2\frac{\langle\hat r_{\rm V}'^\dagger\hat t_{\rm H}^\dagger\hat t_{\rm H}\hat r_{\rm V}'\rangle+\langle\hat r_{\rm V}^\dagger\hat t_{\rm H}'^\dagger\hat t_{\rm H}'\hat r_{\rm V}\rangle}{\langle\hat a_{\rm L}^\dagger\hat a_{\rm L}\rangle\,\langle\hat a_{\rm R}^\dagger\hat a_{\rm R}\rangle}=\frac{\langle\hat a_{\rm R}'^\dagger\hat a_{\rm L}^\dagger\hat a_{\rm L}\hat a_{\rm R}'^\dagger\rangle}{\langle\hat a_{\rm L}^\dagger\hat a_{\rm L}\rangle\,\langle\hat a_{\rm R}^\dagger\hat a_{\rm R}\rangle}=\epsilon_{\rm d}, \tag{9}$$

where $\hat t_{\rm H}'$ ($\hat r_{\rm V}'$) is the transmitted (reflected) distinguishable output mode.

By subtracting the visibility reduction due to background detection events ($0.034(8)\,\%$), single-photon impurity ($0.036(10)\,\%$), and imperfect optical elements ($0.005(7)\,\%$) from the measured HOM interference visibility $V(0)=$

	T_d = 80 ns	T_d = 200 ns	T_d = 300 ns	T_d =400 ns
Percentage of the single-photon wave-packet in T_d	62%	94%	99%	99.9%
Visibility reduction due to background detection events	0.034(8)%	0.051(7)%	0.075(8)%	0.101(8)%
Visibility reduction due to single-photon impurity	0.036(10)%	0.061(10)%	0.071(11)%	0.077(11)%
Visibility reduction due to imperfect optical elements	0.005(7)%	0.005(7)%	0.005(7)%	0.005(7)%
HOM visibility	99.87(8)%	99.43(9)%	99.13(10)%	98.91(11)%
Photon indistinguishability	99.94(8)%	99.55(9)%	99.28(10)%	99.09(11)%

Supplementary Table 1. Photon interference visibility and indistinguishability for different detection window T_d

99.87(8) %, a photon distinguishability of $\epsilon_{\rm d} = 0.06(8)\,\%$ is extracted. We estimate that the indistinguishability of our Rydberg single-photons source is $1 - \epsilon_{\rm d} = 99.94(8)\,\%$.

We also analyze the HOM data for 200 ns detection window. Besides finite photon indistinguishability, the HOM visibility is also reduced by the following sources: 0.051(7) % from the background, 0.061(10) % from single-photon impurity, and 0.005(7) % from the imperfect optical elements. From the measured HOM visibility $V(0) = 99.43(9)\,\%$, we extract a indistinguishability of $1 - \epsilon_{\rm d} = 99.55(9)\,\%$ for 200 ns detection window. We summarize the results in Supplementary Table 1.

III. SUPPLEMENTARY NOTE 3: HIGH FIDELITY PHOTON-PHOTON QUANTUM LOGIC GATE

High-fidelity photon-photon quantum logic gate lies at the heart of scalable quantum architectures such as photonic quantum computers[1–3] and all-optical quantum repeaters[4,5]. The simplest quantum operation relying on photon-photon interactions is the two-photon quantum logic gate, e.g., the CNOT gate. Since the strong single-photon-level nonlinearity is difficult to achieve, only a few experiments that harness matter-light interactions to implement two-photon gates have been demonstrated[6,7]. Most existing protocols for two-photon quantum logic gates are based on quantum interference, single-photon detection and post-selection[8–19].

However, realizing high-fidelity two-photon quantum logic gates remains elusive due to the lack of perfect single-photon sources. For example, to realize a quantum logic gate with fidelity approaching 99 % using the interference-based CNOT gate protocol, the minimum requirements on the single-photon source are $g^{(2)}(0) < 7 \times 10^{-3}$ and indistinguishability higher than 99 %. To date, single-photon source that simultaneously reaches such demanding goals on purity and indistinguishability has not been demonstrated. Physical platforms that hold the promise for generating such near-perfect single photons include quantum dots[20–23], spontaneous parametric down-conversion[24–26], spontaneous four-wave mixing[27,28], nitrogen-vacancy centers[29–31], neutral atoms[32–43], trapped ions[44–47], etc. With a 80 ns detection window, our Rydberg single-photon source shows $g^{(2)}(0) = 4.5(8) \times 10^{-4}$ and indistinguishability of 99.94(8) %, and results in a photon-photon quantum logic gate with a high fidelity. Supplementary Table 2 summarizes the experiments that demonstrated linear-optical gates over the past two decades, and Supplementary Figure 5 displays the achieved truth table and entangling gate infidelities on a logarithmic scale. The infidelities in our work are suppressed by more than a order of magnitude compared to previous studies.

In the remainder of this section, we analyze the detailed implementation of the CNOT gate, and investigate the major sources of error that affect the truth table fidelity in the experiment. As shown in Supplementary Figure 6, the CNOT gate establishes quantum correlations between the control and target photons by interfering them at a

No.	Reference	Truth table fidelity	Entangling gate fidelity	Intrinsic success probability	Single-photon source
(1)	Phys. Rev. Lett. 88, 257902 (2002)[8]	0.83	–	1/4	SPDC
(2)	Nature 426, 264 (2003)[11]	0.84	0.87(8)	1/9	SPDC
(3)	Phys. Rev. A 68, 032316 (2003)[48]	0.80	–	1/4	SPDC
(4)	Phys. Rev. Lett. 93, 080502 (2004)[49]	0.90	0.73	1/9	SPDC
(5)	Phys. Rev. Lett. 93, 240501 (2004)[50]	0.84	0.81	1/4	SPDC
(6)	Phys. Rev. Lett. 93, 020504 (2004)[51]	0.79	0.81	1/16	SPDC
(7)	Phys. Rev. Lett. 95, 210504 (2005)[12]	0.872(1)	–	1/9	SPDC
(8)	Phys. Rev. Lett. 95, 210506 (2005)[14]	0.87	–	1/9	SPDC
(9)	Phys. Rev. Lett. 95, 210505 (2005)[13]	0.818	0.878(6)	1/9	SPDC
(10)	Phys. Rev. Lett. 94, 030501 (2005)[52]	0.79(5)	–	1/4	SPDC
(11)	Phys. Rev. Lett. 98, 170502 (2007)[53]	0.90	–	1/8	SPDC
(12)	Science 320, 646 (2008)[54]	0.943(2)	0.92	1/9	SPDC
(13)	Phys. Rev. Lett. 100, 133603 (2008)[55]	0.87	0.80(2)	1/9	FWM
(14)	IEEE J. Sel. Top. Quantum Electron. 15, 1685 (2009)[56]	0.891(3)	–	1/9	FWM
(15)	Proc. Natl Acad. Sci. USA 107, 20869 (2010)[57]	0.72(5)	0.575(27)	1/9	SPDC
(16)	Nat. Commun. 2, 566 (2011)[15]	0.940(4)	0.939(8)	1/9	SPDC
(17)	Phys. Rev. Lett. 106, 013602 (2011)[58]	0.95	–	1/9	SPDC
(18)	Proc. Natl Acad. Sci. USA 108, 10067 (2011)[59]	0.88(2)	–	1/16	SPDC
(19)	Nat. Photon. 6, 45 (2012)[60]	0.873(1)	0.947(2)	1/9	SPDC
(20)	Appl. Phys. Lett. 100, 211103 (2012)[16]	0.75	–	1/9	SPDC
(21)	Nat. Nanotechnol. 8, 213 (2013)[17]	0.85(6)	–	1/9	QDs
(22)	Phys. Rev. Lett. 110, 250501 (2013)[61]	0.730(16)	0.710(36)	1/9	QDs
(23)	Science 349, 711 (2015)[62]	0.930(3)	0.943(4)	1/16	SPDC
(24)	Nat. Photon. 12, 534 (2018)[18]	0.9885(6)	–	1/64	FWM
(25)	npj Quantum Inf. 4, 13 (2018)[63]	0.838(26)	0.76(5)	1/4	SPDC
(26)	Opt. Mater. Express 9, 2318 (2019)[64]	0.980(6)	–	1/9	SPDC
(27)	Phys. Rev. Lett. 126, 130501 (2021)[19]	0.944	0.93(3)	1/9	SPDC
(28)	Phys. Rev. Lett. 126, 140501 (2021)[65]	0.886(21)	0.834(24)	1/8	QDs
(29)	Phys. Rev. Lett. 128, 060501 (2022)[66]	0.89(2)	0.90(2)	1/9	SPDC
(30)	**This work**	**0.9984(3)**	**0.9969(4)**	**1/9**	**Rydberg**

Supplementary Table 2. A quantitative comparison of linear-optical CNOT gates, to the best of our knowledge. SPDC: spontaneous parametric down-conversion; FWM: four-wave mixing; QDs: quantum dots.

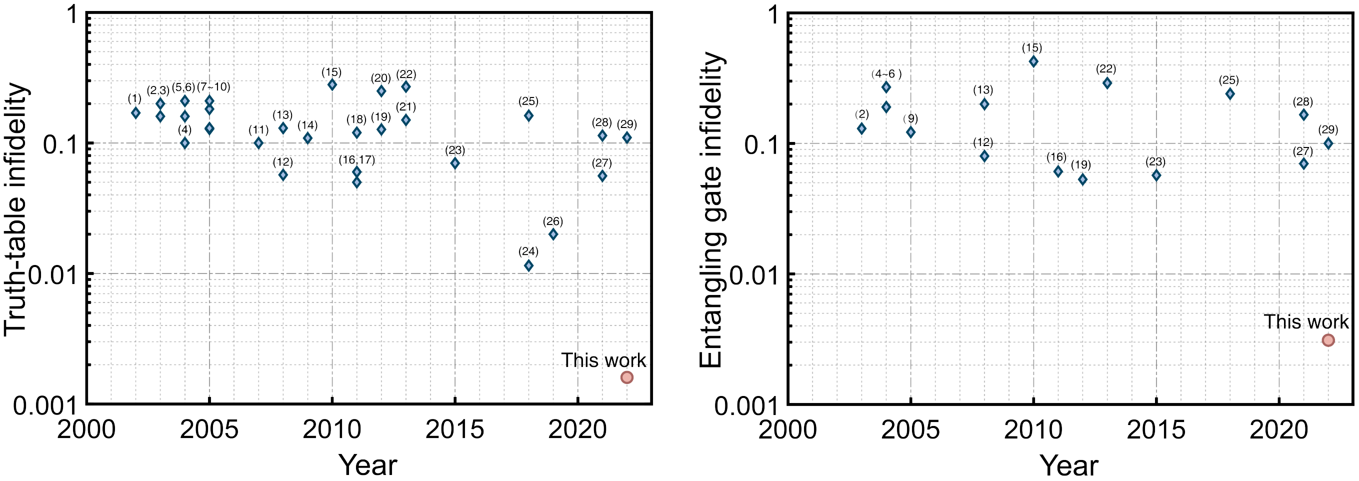

Supplementary Figure 5. A comparison of state-of-the-art truth table and entangling gate infidelities in linear-optical quantum logic gate experiments.

partial polarization beam splitter (PPBS), which has a reflectivity of 1/3 (1) for horizontally (vertically) polarized photons. For an input state of $|\mathrm{HH}\rangle$, unlike a HOM interferometer which completely eliminates $|\mathrm{HH}\rangle$ state at the output, the PPBS-based photon-photon interferometer generates an output state with π-phase shift : $-1/3\,|\mathrm{HH}\rangle$, due to the unbalanced destructive interference caused by the partial reflection. By encoding the qubits with scheme shown in Supplementary Table 3 and combing the controlled π-phase shift mechanism with two more PPBSs for amplitude compensation, the two-photon CNOT gate operation can be executed[11–19].

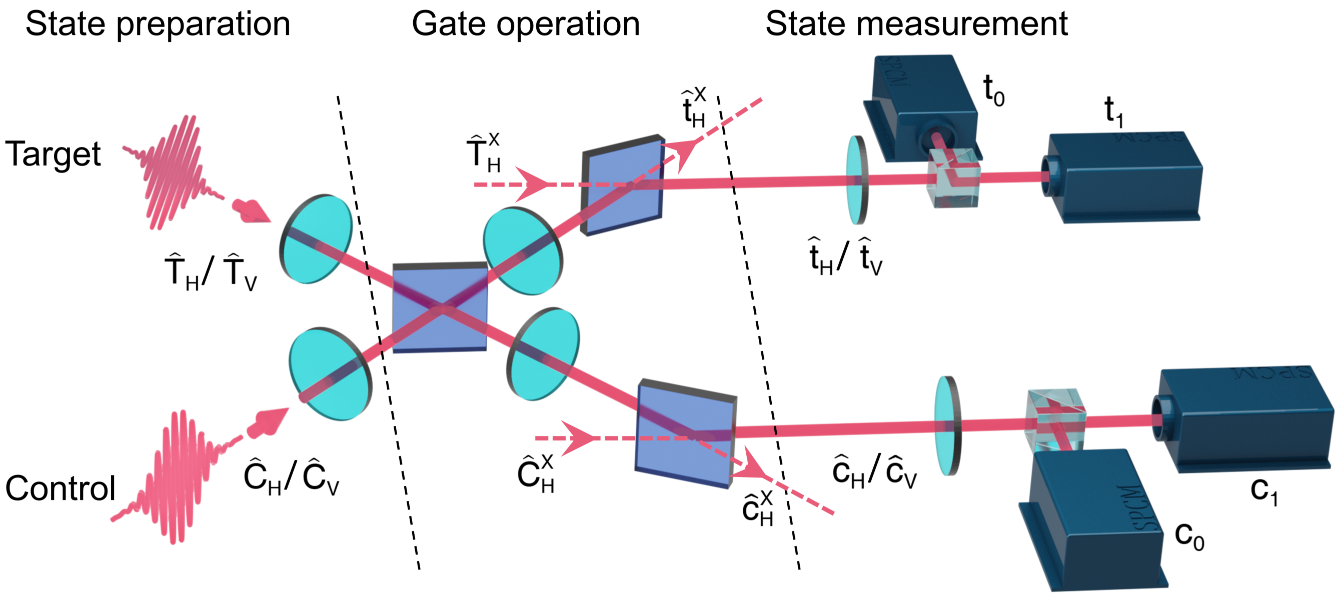

Supplementary Figure 6. Schematic illustration of the CNOT gate apparatus. The CNOT gate experimental setup consists of state preparation, the gate operation and the state measurement.

In order to analyze various gate errors, we model the CNOT gate protocol in detail. As shown in Supplementary Figure 6, the gate operation transforms the input optical modes $\hat{C}_{\mathrm{H}}$, $\hat{C}_{\mathrm{V}}$, $\hat{T}_{\mathrm{H}}$ and $\hat{T}_{\mathrm{V}}$ to the output modes $\hat{c}_{\mathrm{H}}$, $\hat{c}_{\mathrm{V}}$, $\hat{t}_{\mathrm{H}}$ and $\hat{t}_{\mathrm{V}}$, where symbols $\hat{C}$ ($\hat{T}$) and $\hat{c}$ ($\hat{t}$) represent the input and output modes of the control (target) arm, and the subscript H (V) indicates a horizontally (vertically) polarized mode. To balance the amplitudes of the output modes, two more PPBSs and HWPs are employed after the two-photon interference. To describe the effects of these two PPBSs, ancillary input and output modes of $\hat{C}_{\mathrm{H}}^{\mathrm{X}}$ ($\hat{T}_{\mathrm{H}}^{\mathrm{X}}$) and $\hat{c}_{\mathrm{H}}^{\mathrm{X}}$ ($\hat{t}_{\mathrm{H}}^{\mathrm{X}}$) are introduced. The detailed input-output transformation is described by

$$\begin{pmatrix} \hat{c}_{\mathrm{H}} \\ \hat{t}_{\mathrm{H}} \\ \hat{c}_{\mathrm{V}} \\ \hat{t}_{\mathrm{V}} \\ \hat{c}_{\mathrm{H}}^{\mathrm{X}} \\ \hat{t}_{\mathrm{H}}^{\mathrm{X}} \end{pmatrix} = \begin{pmatrix} 0 & 0 & \sqrt{1/3} & 0 & -\sqrt{2/3} & 0 \\ 0 & 0 & 0 & \sqrt{1/3} & 0 & -\sqrt{2/3} \\ \sqrt{1/3} & -\sqrt{2/3} & 0 & 0 & 0 & 0 \\ \sqrt{2/3} & \sqrt{1/3} & 0 & 0 & 0 & 0 \\ 0 & 0 & \sqrt{2/3} & 0 & \sqrt{1/3} & 0 \\ 0 & 0 & 0 & \sqrt{2/3} & 0 & \sqrt{1/3} \end{pmatrix} \begin{pmatrix} \hat{C}_{\mathrm{H}} \\ \hat{T}_{\mathrm{H}} \\ \hat{C}_{\mathrm{V}} \\ \hat{T}_{\mathrm{V}} \\ \hat{C}_{\mathrm{H}}^{\mathrm{X}} \\ \hat{T}_{\mathrm{H}}^{\mathrm{X}} \end{pmatrix}. \tag{10}$$

	Control		Target	
	$\vert 0\rangle$	$\vert 1\rangle$	$\vert 0\rangle$	$\vert 1\rangle$
Input qubits	$\hat{C}_{\mathrm{V}}$	$-\hat{C}_{\mathrm{H}}$	$\left(\hat{T}_{\mathrm{H}}+\hat{T}_{\mathrm{V}}\right)/\sqrt{2}$	$\left(\hat{T}_{\mathrm{V}}-\hat{T}_{\mathrm{H}}\right)/\sqrt{2}$
Output qubits	$\hat{c}_{\mathrm{H}}$	$-\hat{c}_{\mathrm{V}}$	$(\hat{t}_{\mathrm{H}}+\hat{t}_{\mathrm{V}})/\sqrt{2}$	$(\hat{t}_{\mathrm{H}}-\hat{t}_{\mathrm{V}})/\sqrt{2}$

Supplementary Table 3. The encoding scheme for input and output qubits. According to the encoding scheme in the main text, we define $\hat{C}_0\backslash\hat{C}_1 = \hat{C}_{\mathrm{V}}\backslash - \hat{C}_{\mathrm{H}}$ and $\hat{T}_0\backslash\hat{T}_1 = \hat{T}_{\mathrm{D}}\backslash - \hat{T}_{\mathrm{A}} = (\hat{T}_{\mathrm{V}} \pm \hat{T}_{\mathrm{H}})/\sqrt{2}$ as the input qubits. The output qubits are encoded as $\hat{c}_0\backslash\hat{c}_1 = \hat{c}_{\mathrm{H}}\backslash - \hat{c}_{\mathrm{V}}$ and $\hat{t}_0\backslash\hat{t}_1 = \hat{t}_{\mathrm{D}}\backslash\hat{t}_{\mathrm{A}} = (\hat{t}_{\mathrm{H}} \pm \hat{t}_{\mathrm{V}})/\sqrt{2}$.

Assisted by the non-classical interference between two indistinguishable single photons, the transformation in Sup-

plementary Equation (10) establishes quantum correlations between the control and target photonic qubits. After the interference, two single photons could end up in three scenarios: (i) a single photon occupies one of the control modes and the other occupies one of the target modes, (ii) both single photons appear in the control or target modes, (iii) one or two single photons leak to the ancillary modes. The coincidence detection events between the control and target output qubits only occur in scenario (i). By post selecting these events, a CNOT operation on the input photons is implemented in terms of the encoding displayed in Supplementary Table 3, and the operation can be described as

$$\left(\hat{C}_0^\dagger \hat{T}_0^\dagger \;\; \hat{C}_0^\dagger \hat{T}_1^\dagger \;\; \hat{C}_1^\dagger \hat{T}_0^\dagger \;\; \hat{C}_1^\dagger \hat{T}_1^\dagger \right) \rightarrow \frac{1}{3} \left(\hat{c}_0^\dagger \hat{t}_0^\dagger \;\; \hat{c}_0^\dagger \hat{t}_1^\dagger \;\; \hat{c}_1^\dagger \hat{t}_0^\dagger \;\; \hat{c}_1^\dagger \hat{t}_1^\dagger \right) \begin{pmatrix} 1 & 0 & 0 & 0 \\ 0 & 1 & 0 & 0 \\ 0 & 0 & 0 & 1 \\ 0 & 0 & 1 & 0 \end{pmatrix}. \tag{11}$$

In addition to the truth table in computational ZZ basis, we also measure the truth table in complementary XX basis, defined by $|\pm\rangle_{\mathrm{C}} = (|0\rangle_{\mathrm{C}} \pm |1\rangle_{\mathrm{C}})/\sqrt{2}$ for the control qubit and $|\pm\rangle_{\mathrm{T}} = (|0\rangle_{\mathrm{T}} \pm |1\rangle_{\mathrm{T}})/\sqrt{2}$ for the target qubit. The truth table fidelities are given by $F_{\mathrm{CNOT}}^{\mathrm{ZZ}} = 99.84(3)\,\%$ and $F_{\mathrm{CNOT}}^{\mathrm{XX}} = 99.81(3)\,\%$. In the following, we analyze the major sources of error that contribute to the infidelities: background detection events, single-photon impurity, imperfect photon indistinguishability, and imperfections of optical components.

Infidelities from background detection events. One of the error contributions comes from the background detection events, which are dominated by the SPCM dark counts. The probability of error for each combination of input-output states are approximated to the first-order and shown in Supplementary Table 4. Several notations are introduced to denote various probabilities in each experiment cycle. When the input state is $|ij\rangle$, $p_{mn}^{(ij)}$ represents the probability of coincidence between photons in polarization states of $|m\rangle_{\mathrm{c}}$ and $|n\rangle_{\mathrm{t}}$, $p_{\mathrm{c}_m}^{(ij)}$ ($p_{\mathrm{t}_n}^{(ij)}$) denotes the probability of detecting a single photon in $|m\rangle_{\mathrm{c}}$ ($|n\rangle_{\mathrm{t}}$), and $p_{\mathrm{c}_m}^{\mathrm{B}(ij)}$ ($p_{\mathrm{t}_n}^{\mathrm{B}(ij)}$) is the background detection probability of SPCM corresponding to output state $|m\rangle_{\mathrm{c}}$ ($|n\rangle_{\mathrm{t}}$), where $i, j, m, n = 0, 1$ in ZZ basis and $i, j, m, n = +, -$ in XX basis. We estimate that the errors due to the background detection events add up to infidelities of $\Delta F_{\mathrm{bg}}^{\mathrm{ZZ}} = 0.088(4)\,\%$ for ZZ basis and $\Delta F_{\mathrm{bg}}^{\mathrm{XX}} = 0.077(4)\,\%$ for XX basis.

Infidelities from single-photon impurity. As a result of the single-photon impurity, there are residual multi-photon components from our single-photon source, which also reduce the truth table fidelity. In the measurement of truth table in ZZ basis, this error mechanism depends on the state of control qubit. When the control qubit is in state $|0\rangle_{\mathrm{C}}$, the photons from mode $\hat{C}_0$ can only end up in mode $\hat{c}_0$ and do not contribute any coincidences. However, the mode $\hat{T}_{j=0,1}$ is allowed to transform to both $\hat{c}_1$ and $\hat{t}_j$ modes. Multi-photon events in $\hat{T}_j$ mode result in an error in the element $|0\rangle_{\mathrm{C}} |j\rangle_{\mathrm{T}} \rightarrow |1\rangle_{\mathrm{c}} |j\rangle_{\mathrm{t}}$ of the truth table

$$\frac{\langle \hat{t}_j^\dagger \hat{c}_1^\dagger \hat{c}_1 \hat{t}_j \rangle_{C=0,T=j}}{\frac{1}{9} \langle \hat{C}_0^\dagger \hat{C}_0 \rangle_{C=0} \langle \hat{T}_j^\dagger \hat{T}_j \rangle_{T=j}} = \frac{\langle \hat{T}_j^\dagger \hat{T}_j^\dagger \hat{T}_j \hat{T}_j \rangle_{T=j}}{\langle \hat{C}_0^\dagger \hat{C}_0 \rangle_{C=0} \langle \hat{T}_j^\dagger \hat{T}_j \rangle_{T=j}} = \frac{\eta_{\mathrm{T}}}{\eta_{\mathrm{C}}} g_{\mathrm{T}}^{(2)}(0) = 6.0(17) \times 10^{-4}. \tag{12}$$

Here, the factor 1/9 is the success probability of the CNOT operation (derived from Supplementary Equation (11)), and the denominator is the probability of two-photon inputs. The η_{C} (η_{T}) is the probability of having a photon in the control (target) arm, and $\eta_{\mathrm{T}}/\eta_{\mathrm{C}} = 2.11(4)$ is measured with coherent laser light. When the control qubit is in state $|1\rangle_{\mathrm{C}}$, photons from input mode $\hat{C}_1$ go to output modes $\hat{c}_1$, $\hat{t}_0$ and $\hat{t}_1$. The multi-photon components from input mode $\hat{C}_1$ or $\hat{T}_j$ lead to an error in the element $|1\rangle_{\mathrm{C}} |j\rangle_{\mathrm{T}} \rightarrow |1\rangle_{\mathrm{c}} |j\rangle_{\mathrm{t}}$ of the truth table

$$\frac{\langle \hat{t}_j^\dagger \hat{c}_1^\dagger \hat{c}_1 \hat{t}_j \rangle_{C=1,T=j}}{\frac{1}{9} \langle \hat{C}_1^\dagger \hat{C}_1 \rangle_{C=1} \langle \hat{T}_j^\dagger \hat{T}_j \rangle_{T=j}} = \frac{\langle \hat{C}_j^\dagger \hat{C}_j^\dagger \hat{C}_j \hat{C}_j \rangle_{C=1} + \langle \hat{T}_j^\dagger \hat{T}_j^\dagger \hat{T}_j \hat{T}_j \rangle_{T=j}}{\langle \hat{C}_1^\dagger \hat{C}_1 \rangle_{C=1} \langle \hat{T}_j^\dagger \hat{T}_j \rangle_{T=j}} = \frac{\eta_{\mathrm{C}}}{\eta_{\mathrm{T}}} g_{\mathrm{C}}^{(2)}(0) + \frac{\eta_{\mathrm{T}}}{\eta_{\mathrm{C}}} g_{\mathrm{T}}^{(2)}(0) = 7.4(21) \times 10^{-4}. \tag{13}$$

Here, $g_{\mathrm{C}}^{(2)}(0) = g_{\mathrm{T}}^{(2)}(0) = 2.9(8) \times 10^{-4}$ is determined in Section I. Considering the gate errors in Supplementary Equations (12) and (13), the infidelity from multi-photon components for ZZ basis is given by

$$\Delta F_{\mathrm{impurity}}^{\mathrm{ZZ}} = \frac{\eta_{\mathrm{C}}}{2\eta_{\mathrm{T}}} g_{\mathrm{C}}^{(2)}(0) + \frac{\eta_{\mathrm{T}}}{\eta_{\mathrm{C}}} g_{\mathrm{T}}^{(2)}(0) = 0.067(19)\,\%. \tag{14}$$

The difference between the measurements of truth tables in XX and ZZ bases is that the polarization bases of the control and target qubits are exchanged. Thus, we can derive that the contributed infidelity for XX basis is

$$\Delta F_{\mathrm{impurity}}^{\mathrm{XX}} = \frac{\eta_{\mathrm{C}}}{\eta_{\mathrm{T}}} g_{\mathrm{C}}^{(2)}(0) + \frac{\eta_{\mathrm{T}}}{2\eta_{\mathrm{C}}} g_{\mathrm{T}}^{(2)}(0) = 0.044(12)\,\%. \tag{15}$$

ZZ basis				
Output \ Input	$\vert 00\rangle$	$\vert 01\rangle$	$\vert 10\rangle$	$\vert 11\rangle$
$\vert 00\rangle$	0	$\frac{p_{c_0}^{(01)} p_{t_0}^{B(01)}}{p_{01}^{(01)}}$ $= 0.8(1) \times 10^{-4}$	$\frac{p_{t_0}^{(10)} p_{c_0}^{B(10)}}{p_{11}^{(10)}}$ $= 1.9(3) \times 10^{-4}$	$\frac{p_{t_0}^{(11)} p_{c_0}^{B(11)}}{p_{10}^{(11)}}$ $= 0.9(1) \times 10^{-4}$
$\vert 01\rangle$	$\frac{p_{c_0}^{(00)} p_{t_1}^{B(00)}}{p_{00}^{(00)}}$ $= 1.0(2) \times 10^{-4}$	0	$\frac{p_{t_1}^{(10)} p_{c_0}^{B(10)}}{p_{11}^{(10)}}$ $= 0.7(1) \times 10^{-4}$	$\frac{p_{t_1}^{(11)} p_{c_0}^{B(11)}}{p_{10}^{(11)}}$ $= 2.4(4) \times 10^{-4}$
$\vert 10\rangle$	$\frac{p_{c_1}^{(00)} p_{t_0}^{B(00)} + p_{t_0}^{(00)} p_{c_1}^{B(00)}}{p_{00}^{(00)}}$ $= 4.9(5) \times 10^{-4}$	$\frac{p_{c_1}^{(01)} p_{t_0}^{B(01)}}{p_{01}^{(01)}}$ $= 1.5(3) \times 10^{-4}$	$\frac{p_{c_1}^{(10)} p_{t_0}^{B(10)} + p_{t_0}^{(10)} p_{c_1}^{B(10)}}{p_{11}^{(10)}}$ $= 6.0(6) \times 10^{-4}$	0
$\vert 11\rangle$	$\frac{p_{c_1}^{(00)} p_{t_1}^{B(00)}}{p_{00}^{(00)}}$ $= 1.8(3) \times 10^{-4}$	$\frac{p_{c_1}^{(01)} p_{t_1}^{B(01)} + p_{t_1}^{(01)} p_{c_1}^{B(01)}}{p_{01}^{(01)}}$ $= 6.1(5) \times 10^{-4}$	0	$\frac{p_{c_1}^{(11)} p_{t_1}^{B(11)} + p_{t_1}^{(11)} p_{c_1}^{B(11)}}{p_{10}^{(11)}}$ $= 7.4(7) \times 10^{-4}$
XX basis				
Output \ Input	$\vert ++\rangle$	$\vert +-\rangle$	$\vert -+\rangle$	$\vert --\rangle$
$\vert ++\rangle$	0	$\frac{p_{c_+}^{(+-)} p_{t_+}^{B(+-)}}{p_{--}^{(+-)}}$ $= 2.4(4) \times 10^{-4}$	$\frac{p_{t_+}^{(-+)} p_{c_+}^{B(-+)}}{p_{-+}^{(-+)}}$ $= 2.2(3) \times 10^{-4}$	$\frac{p_{c_+}^{(--)} p_{t_+}^{B(--)}}{p_{+-}^{(--)}}$ $= 1.7(3) \times 10^{-4}$
$\vert +-\rangle$	$\frac{p_{c_+}^{(++)} p_{t_-}^{B(++)} + p_{t_-}^{(++)} p_{c_+}^{B(++)}}{p_{++}^{(++)}}$ $= 1.5(2) \times 10^{-4}$	$\frac{p_{c_+}^{(+-)} p_{t_-}^{B(+-)} + p_{t_-}^{(+-)} p_{c_+}^{B(+-)}}{p_{--}^{(+-)}}$ $= 6.0(6) \times 10^{-4}$	$\frac{p_{t_-}^{(-+)} p_{c_+}^{B(-+)}}{p_{-+}^{(-+)}}$ $= 0.8(1) \times 10^{-4}$	0
$\vert -+\rangle$	$\frac{p_{t_+}^{(++)} p_{c_-}^{B(++)}}{p_{++}^{(++)}}$ $= 1.8(3) \times 10^{-4}$	$\frac{p_{c_-}^{(+-)} p_{t_+}^{B(+-)}}{p_{--}^{(+-)}}$ $= 2.1(3) \times 10^{-4}$	0	$\frac{p_{c_-}^{(--)} p_{t_+}^{B(--)}}{p_{+-}^{(--)}}$ $= 2.1(4) \times 10^{-4}$
$\vert --\rangle$	$\frac{p_{t_-}^{(++)} p_{c_-}^{B(++)}}{p_{++}^{(++)}}$ $= 0.6(1) \times 10^{-4}$	0	$\frac{p_{c_-}^{(-+)} p_{t_-}^{B(-+)} + p_{t_-}^{(-+)} p_{c_-}^{B(-+)}}{p_{-+}^{(-+)}}$ $= 2.1(2) \times 10^{-4}$	$\frac{p_{c_-}^{(--)} p_{t_-}^{B(--)} + p_{t_-}^{(--)} p_{c_-}^{B(--)}}{p_{+-}^{(--)}}$ $= 7.5(7) \times 10^{-4}$

Supplementary Table 4. Error contribution of background detection events to each element of the truth tables for ZZ and XX bases.

Infidelities from photon distinguishability. To analyze the infidelity due to imperfect photon indistinguishability, similar to Section II, we introduce additional modes $\hat{T}'_{j=0,1}$ to characterize the distinguishable components

between the control and target photons. The target modes can be written as $\sqrt{1-\epsilon_{\mathrm{d}}}\hat{T}_j + \sqrt{\epsilon_{\mathrm{d}}}\hat{T}'_j$, where the distinguishability ϵ_{d} is defined in Section II. In the measurement of the truth table in ZZ basis, the two-photon interference occurs only when the control qubit is in state $|1\rangle_{\mathrm{C}}$. The existence of the distinguishable input mode $\hat{T}'_j$ deteriorates the interference and causes an error in the element $|1\rangle_{\mathrm{C}}|j\rangle_{\mathrm{T}} \to |1\rangle_{\mathrm{c}}|j\rangle_{\mathrm{t}}$ of the truth table

$$9\frac{\langle \hat{t}_j^\dagger \hat{c}_1^\dagger \hat{c}_1 \hat{t}_j + \hat{t}_j'^\dagger \hat{c}_1^\dagger \hat{c}_1 \hat{t}'_j + \hat{t}_j^\dagger \hat{c}_1'^\dagger \hat{c}'_1 \hat{t}_j\rangle}{\langle \hat{C}_1^\dagger \hat{C}_1\rangle \langle \hat{T}_j^\dagger \hat{T}_j + \hat{T}_j'^\dagger \hat{T}'_j\rangle} \approx 2\epsilon_{\mathrm{d}} = 0.0012(16), \tag{16}$$

where $\hat{c}'_1$ and $\hat{t}'_j$ are the distinguishable output modes. By averaging the errors described in Supplementary Equation (16) when the control qubit is in $|1\rangle_{\mathrm{C}}$ and the zero errors when the control qubit is in $|0\rangle_{\mathrm{C}}$, the distinguishability contributed infidelity is given by $\Delta F_{\mathrm{d}}^{\mathrm{ZZ}} = \epsilon_{\mathrm{d}} = 0.06(8)\,\%$. For XX basis, this contribution is the same.

Infidelities from imperfect optical elements. We analyze the imperfections of optical components in state preparation, gate operation and state measurement stages separately. In the state preparation stage, considering the imperfections of HWPs and the finite resolutions of our polarization calibrations (limited by the polarimeter), ϵ_{C} (ϵ_{T}) is introduced to characterize the polarization state error of the control (target) qubit. Therefore, we redefine the encoding of the input qubits (see Supplementary Table 3) as

$$\begin{pmatrix} \hat{C}_0 \\ \hat{T}_0 \\ \hat{C}_1 \\ \hat{T}_1 \end{pmatrix} = \begin{pmatrix} \frac{\epsilon_{\mathrm{C}}}{2} & 0 & -1 & 0 \\ 0 & \frac{1}{\sqrt{2}}\left(1+\frac{\epsilon_{\mathrm{T}}}{2}\right) & 0 & -\frac{1}{\sqrt{2}}\left(1-\frac{\epsilon_{\mathrm{T}}}{2}\right) \\ 1 & 0 & \frac{\epsilon_{\mathrm{C}}}{2} & 0 \\ 0 & \frac{1}{\sqrt{2}}\left(1-\frac{\epsilon_{\mathrm{T}}}{2}\right) & 0 & \frac{1}{\sqrt{2}}\left(1+\frac{\epsilon_{\mathrm{T}}}{2}\right) \end{pmatrix} \begin{pmatrix} \hat{C}_{\mathrm{H}} \\ \hat{T}_{\mathrm{H}} \\ \hat{C}_{\mathrm{V}} \\ \hat{T}_{\mathrm{V}} \end{pmatrix}. \tag{17}$$

In the gate operation stage, the main imperfections come from the three PPBSs. We denote the reflectivity of each PPBS as $(1-\epsilon_{\mathrm{H}_i})/3$ for horizontally polarized components instead of the ideal value 1/3, and $(1-\epsilon_{\mathrm{V}_i}^2)$ for vertically polarized components instead of 1, where $i=0$ corresponds to the PPBS for two-photon interference, and $i=1,2$ correspond to the PPBSs in the control and target arms, respectively. Combining with Supplementary Equation (17), we derive the transformation matrix of the CNOT gate approximated to the first-order as

$$\mathbf{U}_{\mathrm{CNOT}} \approx \begin{pmatrix} 1-\frac{\epsilon_{\mathrm{H}_0}}{4}-\frac{\epsilon_{\mathrm{H}_1}}{2}-\frac{\epsilon_{\mathrm{H}_2}}{4} & \frac{\epsilon_{\mathrm{H}_0}}{4}-\frac{\epsilon_{\mathrm{H}_2}}{4}+\frac{\epsilon_{\mathrm{T}}}{2} & \frac{\epsilon_{\mathrm{V}_0}}{\sqrt{2}}+\frac{\epsilon_{\mathrm{C}}}{2} & \frac{\epsilon_{\mathrm{V}_0}}{\sqrt{2}} \\ \frac{\epsilon_{\mathrm{H}_0}}{4}-\frac{\epsilon_{\mathrm{H}_2}}{4}-\frac{\epsilon_{\mathrm{T}}}{2} & 1-\frac{\epsilon_{\mathrm{H}_0}}{4}-\frac{\epsilon_{\mathrm{H}_1}}{2}-\frac{\epsilon_{\mathrm{H}_2}}{4} & -\frac{\epsilon_{\mathrm{V}_0}}{\sqrt{2}} & -\frac{\epsilon_{\mathrm{V}_0}}{\sqrt{2}}+\frac{\epsilon_{\mathrm{C}}}{2} \\ \frac{\epsilon_{\mathrm{V}_0}}{\sqrt{2}} & -\frac{\epsilon_{\mathrm{V}_0}}{\sqrt{2}}-\frac{\epsilon_{\mathrm{C}}}{2} & -\frac{5\epsilon_{\mathrm{H}_0}}{4}-\frac{\epsilon_{\mathrm{H}_2}}{4}-\frac{\epsilon_{\mathrm{T}}}{2} & 1+\frac{3\epsilon_{\mathrm{H}_0}}{4}-\frac{\epsilon_{\mathrm{H}_2}}{4} \\ \frac{\epsilon_{\mathrm{V}_0}}{\sqrt{2}}-\frac{\epsilon_{\mathrm{C}}}{2} & -\frac{\epsilon_{\mathrm{V}_0}}{\sqrt{2}} & 1+\frac{3\epsilon_{\mathrm{H}_0}}{4}-\frac{\epsilon_{\mathrm{H}_2}}{4} & -\frac{5\epsilon_{\mathrm{H}_0}}{4}-\frac{\epsilon_{\mathrm{H}_2}}{4}+\frac{\epsilon_{\mathrm{T}}}{2} \end{pmatrix}. \tag{18}$$

In the state measurement stage, the imperfections of HWP and PBS in the control (target) arm cause polarization crosstalk between two output control (target) modes. The single photons in mode $\hat{c}_{1-j}$ ($\hat{t}_{1-j}$) could end up in the SPCM corresponding to mode $\hat{c}_j$ ($\hat{t}_j$) with an error probability denoted as $\epsilon_{\mathrm{c}_j}^2$ ($\epsilon_{\mathrm{t}_j}^2$), where $j=0,1$. The above-mentioned imperfections contribute infidelities of

$$\Delta F_{\mathrm{optics}}^{\mathrm{ZZ}} = \epsilon_{\mathrm{V}_0}^2 + \frac{13\epsilon_{\mathrm{H}_0}^2 + 4\epsilon_{\mathrm{H}_0}\epsilon_{\mathrm{H}_2} + \epsilon_{\mathrm{H}_2}^2}{16} + \frac{\epsilon_{\mathrm{C}}^2+\epsilon_{\mathrm{T}}^2}{4} + \frac{\epsilon_{\mathrm{c}_0}^2+\epsilon_{\mathrm{c}_1}^2+\epsilon_{\mathrm{t}_0}^2+\epsilon_{\mathrm{t}_1}^2}{2} = 0.0064(1)\,\% \tag{19}$$

for ZZ basis, and

$$\Delta F_{\mathrm{optics}}^{\mathrm{XX}} = \epsilon_{\mathrm{V}_0}^2 + \frac{13\epsilon_{\mathrm{H}_0}^2 + 4\epsilon_{\mathrm{H}_0}\epsilon_{\mathrm{H}_1} + \epsilon_{\mathrm{H}_1}^2}{16} + \frac{\epsilon_{\mathrm{C}}^2+\epsilon_{\mathrm{T}}^2}{4} + \frac{\epsilon_{\mathrm{c}_0}^2+\epsilon_{\mathrm{c}_1}^2+\epsilon_{\mathrm{t}_0}^2+\epsilon_{\mathrm{t}_1}^2}{2} = 0.0064(1)\,\% \tag{20}$$

for XX basis, where $\epsilon_{\mathrm{V}_0}=0.0080(1)$, $\epsilon_{\mathrm{H}_0}=0.0000(51)$, $\epsilon_{\mathrm{H}_1}=0.0000(60)$, $\epsilon_{\mathrm{H}_2}=0.0000(48)$, $\epsilon_{\mathrm{C}}=0.0000(19)$, $\epsilon_{\mathrm{T}}=0.0000(23)$, $\epsilon_{\mathrm{c}_0}=0.0007(1)$, $\epsilon_{\mathrm{c}_1}=0.0004(2)$, $\epsilon_{\mathrm{t}_0}=0.0005(3)$ and $\epsilon_{\mathrm{t}_1}=0.0010(1)$ are measured by coherent laser light.

Considering various truth table infidelity in ZZ (XX) basis: 0.088(4) % (0.077(4) %) from background detection events, 0.067(19) % (0.044(12) %) from single-photon impurity, 0.06(8) % from photon distinguishability and 0.0064(1) % (0.0064(1) %) from imperfect optical elements, we expect a truth table fidelity of 99.78(8) % (99.82(8) %), for ZZ (XX) basis, which is in good agreement with the measured value of 99.84(3) % (99.81(3) %). We also study the gate performance with 200 ns detection window and find a truth table fidelity of $F_{\mathrm{CNOT}}^{\mathrm{ZZ}} = 99.53(3)\,\%$ ($F_{\mathrm{CNOT}}^{\mathrm{XX}} = 99.40(3)\,\%$). To further improve the gate fidelity, one can increase the single-photon efficiency to get a better signal-to-background ratio, use a smaller atomic sample and higher principal quantum number to suppress multi-photon events, and apply quantum optimal control techniques to optimize photon indistinguishability. With these improvements, it is possible to achieve CNOT gate fidelity above 99.9 % in our system.

In our current experiment, the limitations of success probability come from the intrinsic gate efficiency, the single-photon generation efficiency, a series of optical losses and SPCM detection efficiency. Specifically, the intrinsic efficiency is 1/9, and the single-photon generation efficiency in the CNOT experiment is 0.27. Besides, the overall optical transmission for the target (control) qubit is 0.25 (0.13). The detection efficiency of low-noise SPCMs is about 0.58. Moreover, the 80 ns detection window leads to an extra photon loss of 38 %. Considering the above limitations, our gate detection success probability is about 3.3×10^{-5} with an 80 ns detection window, and the success rate of the gate is up to 100/min with a repetition rate of 50 kHz. We also study the gate performance with 200 ns detection window and find a truth table fidelity of $F^{\mathrm{ZZ}}_{\mathrm{CNOT}} = 99.53(3)\,\%$ ($F^{\mathrm{XX}}_{\mathrm{CNOT}} = 99.40(3)\,\%$). The success probability and success rate of the gate are 7.6×10^{-5} and 230/min, respectively.

We emphasize that the main focus of our work is to achieve high gate fidelity, and we didn't spend efforts on optimizing the efficiencies. Especially, the losses in single-photon efficiency are not fundamental to our experiment and can in principle be alleviated in the future. The single-photon generation efficiency can be further improved, potentially to near unity by incorporating an optical resonator. In the current experiment, no serious efforts have been spent on minimizing the optical losses, which can be alleviated by replacing the optical elements with high transmission and removing some of the optical components used for switching photon paths between different experiments. The detection efficiency can be increased by using superconducting nanowire single-photon detectors (SNSPD). The 38 % photon loss from the 80 ns small detection window is caused by the imperfect interference between the photon components in the edges. We can implement optimal control techniques to the 479 nm read-out field to compensate the imperfect interference and achieve near-unity indistinguishability with the full single-photon wave-packet.

IV. SUPPLEMENTARY NOTE 4: ENTANGLEMENT GENERATION, QUANTUM STATE TOMOGRAPHY AND VIOLATION OF BELL'S INEQUALITY

In this section, the quantum nature of our photon-photon gate is benchmarked by measuring the entangled state fidelity and demonstrating the violation of Bell's inequality. Various error mechanisms that decrease the fidelity and Bell parameter from the ideal values are also thoroughly investigated.

The ability to generate entanglement distinguishes our quantum logic gate from its classical counterpart. The maximally entangled Bell state $|\Psi^-\rangle = (|0\rangle_{\mathrm{c}}|1\rangle_{\mathrm{t}} - |1\rangle_{\mathrm{c}}|0\rangle_{\mathrm{t}})/\sqrt{2}$ is created by inputting a product state of $(|0\rangle_{\mathrm{C}} - |1\rangle_{\mathrm{C}})|1\rangle_{\mathrm{T}}/\sqrt{2}$ to the CNOT gate. The fidelity of the output state ρ relative to $|\Psi^-\rangle$ is

$$F_{\Psi^-} = \langle\Psi^-|\,\rho\,|\Psi^-\rangle = \frac{1}{4}\left(1 - \langle\sigma^{\mathrm{x}}_{\mathrm{c}}\sigma^{\mathrm{x}}_{\mathrm{t}}\rangle - \langle\sigma^{\mathrm{y}}_{\mathrm{c}}\sigma^{\mathrm{y}}_{\mathrm{t}}\rangle - \langle\sigma^{\mathrm{z}}_{\mathrm{c}}\sigma^{\mathrm{z}}_{\mathrm{t}}\rangle\right), \quad (21)$$

where σ^{x}, σ^{y} and σ^{z} are Pauli matrices. We perform a complete quantum state tomography and apply maximum likelihood estimation technique to reconstruct the full density matrix of the output state. The reconstructed density matrix gives a fidelity of $F_{\Psi^-} = 99.69(4)\,\%$, where the standard deviation is given by a Monte Carlo method. The near-unity value for F_{Ψ^-} provides a benchmark for the fidelity of our entangling-gate operation and demonstrates the preparation of high-quality entangled photon pairs, which are critical for a number of quantum applications.

To further verify the entanglement, a Bell's inequality measurement is performed on the output state. Correlations between two output photons are measured at different polarization measurement bases. At linearly polarized bases of $\{\cos\theta_{\mathrm{c}}|0\rangle_{\mathrm{c}} + \sin\theta_{\mathrm{c}}|1\rangle_{\mathrm{c}}, \sin\theta_{\mathrm{c}}|0\rangle_{\mathrm{c}} - \cos\theta_{\mathrm{c}}|1\rangle_{\mathrm{c}}\}$ for control qubit and $\{\cos\theta_{\mathrm{t}}|0\rangle_{\mathrm{t}} + \sin\theta_{\mathrm{t}}|1\rangle_{\mathrm{t}}, \sin\theta_{\mathrm{t}}|0\rangle_{\mathrm{t}} - \cos\theta_{\mathrm{t}}|1\rangle_{\mathrm{t}}\}$ for target qubit, we measure the correlation function of

$$\begin{aligned} E(\theta_{\mathrm{c}},\theta_{\mathrm{t}}) =& \frac{C_{01}(\theta_{\mathrm{c}},\theta_{\mathrm{t}}) + C_{10}(\theta_{\mathrm{c}},\theta_{\mathrm{t}}) - C_{00}(\theta_{\mathrm{c}},\theta_{\mathrm{t}}) - C_{11}(\theta_{\mathrm{c}},\theta_{\mathrm{t}})}{C_{01}(\theta_{\mathrm{c}},\theta_{\mathrm{t}}) + C_{10}(\theta_{\mathrm{c}},\theta_{\mathrm{t}}) + C_{00}(\theta_{\mathrm{c}},\theta_{\mathrm{t}}) + C_{11}(\theta_{\mathrm{c}},\theta_{\mathrm{t}})} \quad (22)\\ =& \frac{p(\theta_{\mathrm{c}},\theta_{\mathrm{t}}+\frac{\pi}{2}) + p(\theta_{\mathrm{c}}+\frac{\pi}{2},\theta_{\mathrm{t}}) - p(\theta_{\mathrm{c}},\theta_{\mathrm{t}}) - p(\theta_{\mathrm{c}}+\frac{\pi}{2},\theta_{\mathrm{t}}+\frac{\pi}{2})}{p(\theta_{\mathrm{c}},\theta_{\mathrm{t}}) + p(\theta_{\mathrm{c}}+\frac{\pi}{2},\theta_{\mathrm{t}}) + p(\theta_{\mathrm{c}},\theta_{\mathrm{t}}+\frac{\pi}{2}) + p(\theta_{\mathrm{c}}+\frac{\pi}{2},\theta_{\mathrm{t}}+\frac{\pi}{2})}, \quad (23) \end{aligned}$$

where C_{ij} is the coincidence rate between SPCMs $\mathrm{c}_{i=0,1}$ and $\mathrm{t}_{j=0,1}$ in Supplementary Figure 6, and $p(\alpha,\beta)$ is the probability of coincidences between output modes $\hat{c}_\alpha = \hat{c}_0\cos\alpha + \hat{c}_1\sin\alpha$ and $\hat{t}_\beta = \hat{t}_0\cos\beta + \hat{t}_1\sin\beta$. We hence obtain the Bell parameter

$$S = E\left(\frac{\pi}{8},0\right) + E\left(\frac{\pi}{8},\frac{\pi}{4}\right) + E\left(-\frac{\pi}{8},0\right) - E\left(-\frac{\pi}{8},\frac{\pi}{4}\right). \quad (24)$$

With $E(\pi/8,0) = 0.706(6)$, $E(\pi/8,\pi/4) = 0.714(6)$, $E(-\pi/8,0) = 0.702(6)$ and $E(-\pi/8,\pi/4) = -0.700(6)$, the Bell parameter is $S = 2.823(12)$, which violates the Clauser-Horne-Shimony-Holt inequality $|S| \leq 2$ by more than 60 standard deviations and unambiguously verifies the photon-photon entanglement created by our CNOT gate.

To understand the reduction of the measured F_{Ψ^-} (S) from its ideal value 1 $(2\sqrt{2})$, we thoroughly analyze major sources of error as follows:

Effects from background detection events. Similar to the analysis in the HOM interference (see Section II) and measurement of truth tables (see Section III), coincidences between single-photon events and background detection events (mainly caused by the SPCM dark counts) increase the coincidence rates between four SPCMs. These background detection events change the values of $\langle\sigma_{\rm c}^{\rm x}\sigma_{\rm t}^{\rm x}\rangle$, $\langle\sigma_{\rm c}^{\rm y}\sigma_{\rm t}^{\rm y}\rangle$ and $\langle\sigma_{\rm c}^{\rm z}\sigma_{\rm t}^{\rm z}\rangle$ by 0.00159(9), 0.00169(9) and 0.00165(10), and $E(\pi/8,0)$, $E(\pi/8,\pi/4)$, $E(-\pi/8,0)$ and $E(-\pi/8,\pi/4)$ by -0.00090(9), -0.00094(8), -0.00099(7) and 0.00139(8), which cause a decrease of F_{Ψ^-} by $\Delta F_{\rm bg}^{\Psi^-}=0.123(4)\,\%$, and S by $\Delta S_{\rm bg}=0.00422(16)$.

Effects from single-photon impurity. For the occupied input modes $(\hat{C}_0-\hat{C}_1)/\sqrt{2}=\hat{C}_{\rm D}$ and $\hat{T}_1=-\hat{T}_{\rm A}$, the multi-photon events increase second-order correlations $\langle\hat{C}_{\rm D}^\dagger\hat{C}_{\rm D}^\dagger\hat{C}_{\rm D}\hat{C}_{\rm D}\rangle$ and $\langle\hat{T}_{\rm A}^\dagger\hat{T}_{\rm A}^\dagger\hat{T}_{\rm A}\hat{T}_{\rm A}\rangle$ from the ideal value of zero. We estimate the impact of these non-zero correlations by calculating

$$\begin{aligned}
p(\alpha,\beta)=\langle\hat{c}_\alpha^\dagger\hat{t}_\beta^\dagger\hat{t}_\beta\hat{c}_\alpha\rangle=\frac{1}{9}\Big[&\langle\hat{C}_{\rm D}^\dagger\hat{C}_{\rm D}\rangle\langle\hat{T}_{\rm A}^\dagger\hat{T}_{\rm A}\rangle\frac{\sin^2(\alpha-\beta)}{2}+\\
&\langle\hat{C}_{\rm D}^\dagger\hat{C}_{\rm D}^\dagger\hat{C}_{\rm D}\hat{C}_{\rm D}\rangle\cos^2\left(\alpha+\frac{\pi}{4}\right)\cos^2\left(\beta+\frac{\pi}{4}\right)+\langle\hat{T}_{\rm A}^\dagger\hat{T}_{\rm A}^\dagger\hat{T}_{\rm A}\hat{T}_{\rm A}\rangle\sin^2\alpha\sin^2\beta\Big],
\end{aligned}\tag{25}$$

$$p({\rm L\backslash R},{\rm L\backslash R})=\langle\hat{c}_{\rm L\backslash R}^\dagger\hat{t}_{\rm L\backslash R}^\dagger\hat{t}_{\rm L\backslash R}\hat{c}_{\rm L\backslash R}\rangle=\frac{1}{36}\Big(\langle\hat{C}_{\rm D}^\dagger\hat{C}_{\rm D}^\dagger\hat{C}_{\rm D}\hat{C}_{\rm D}\rangle+\langle\hat{T}_{\rm A}^\dagger\hat{T}_{\rm A}^\dagger\hat{T}_{\rm A}\hat{T}_{\rm A}\rangle\Big),\tag{26}$$

$$p({\rm L\backslash R},{\rm R\backslash L})=\langle\hat{c}_{\rm L\backslash R}^\dagger\hat{t}_{\rm R\backslash L}^\dagger\hat{t}_{\rm R\backslash L}\hat{c}_{\rm L\backslash R}\rangle=\frac{1}{36}\Big(\langle\hat{C}_{\rm D}^\dagger\hat{C}_{\rm D}^\dagger\hat{C}_{\rm D}\hat{C}_{\rm D}\rangle+\langle\hat{T}_{\rm A}^\dagger\hat{T}_{\rm A}^\dagger\hat{T}_{\rm A}\hat{T}_{\rm A}\rangle+2\langle\hat{C}_{\rm D}^\dagger\hat{C}_{\rm D}\rangle\langle\hat{T}_{\rm A}^\dagger\hat{T}_{\rm A}\rangle\Big),\tag{27}$$

where $\hat{c}_{\rm L\backslash R}=(\hat{c}_0\mp i\hat{c}_1)/\sqrt{2}$ and $\hat{t}_{\rm L\backslash R}=(\hat{t}_0\mp i\hat{t}_1)/\sqrt{2}$. We then derive

$$E(\theta_{\rm c},\theta_{\rm t})=\frac{\langle\hat{C}_{\rm D}^\dagger\hat{C}_{\rm D}\rangle\langle\hat{T}_{\rm A}^\dagger\hat{T}_{\rm A}\rangle\cos2(\theta_{\rm c}-\theta_{\rm t})-\langle\hat{C}_{\rm D}^\dagger\hat{C}_{\rm D}^\dagger\hat{C}_{\rm D}\hat{C}_{\rm D}\rangle\sin2\theta_{\rm c}\sin2\theta_{\rm t}-\langle\hat{T}_{\rm A}^\dagger\hat{T}_{\rm A}^\dagger\hat{T}_{\rm A}\hat{T}_{\rm A}\rangle\cos2\theta_{\rm c}\cos2\theta_{\rm t}}{\langle\hat{C}_{\rm D}^\dagger\hat{C}_{\rm D}\rangle\langle\hat{T}_{\rm A}^\dagger\hat{T}_{\rm A}\rangle+\langle\hat{C}_{\rm D}^\dagger\hat{C}_{\rm D}^\dagger\hat{C}_{\rm D}\hat{C}_{\rm D}\rangle+\langle\hat{T}_{\rm A}^\dagger\hat{T}_{\rm A}^\dagger\hat{T}_{\rm A}\hat{T}_{\rm A}\rangle},\tag{28}$$

$$1+\langle\sigma_{\rm c}^{\rm x}\sigma_{\rm t}^{\rm x}\rangle=1-E\left(\frac{\pi}{4},\frac{\pi}{4}\right)=2\frac{\eta_{\rm C}}{\eta_{\rm T}}g_{\rm C}^{(2)}(0)+\frac{\eta_{\rm T}}{\eta_{\rm C}}g_{\rm T}^{(2)}(0)=0.00088(25),\tag{29}$$

$$1+\langle\sigma_{\rm c}^{\rm y}\sigma_{\rm t}^{\rm y}\rangle=1+\frac{p({\rm L,L})-p({\rm L,R})-p({\rm R,L})+p({\rm R,R})}{p({\rm L,L})+p({\rm L,R})+p({\rm R,L})+p({\rm R,R})}=\frac{\eta_{\rm C}}{\eta_{\rm T}}g_{\rm C}^{(2)}(0)+\frac{\eta_{\rm T}}{\eta_{\rm C}}g_{\rm T}^{(2)}(0)=0.00074(21),\tag{30}$$

$$1+\langle\sigma_{\rm c}^{\rm z}\sigma_{\rm t}^{\rm z}\rangle=1-E(0,0)=\frac{\eta_{\rm C}}{\eta_{\rm T}}g_{\rm C}^{(2)}(0)+2\frac{\eta_{\rm T}}{\eta_{\rm C}}g_{\rm T}^{(2)}(0)=0.00134(38),\tag{31}$$

from which we determine the reduction of Bell parameter and the infidelity of Bell state contributed by the multi-photon events

$$\Delta F_{\rm impurity}^{\Psi^-}\approx\frac{\eta_{\rm C}}{\eta_{\rm T}}g_{\rm C}^{(2)}(0)+\frac{\eta_{\rm T}}{\eta_{\rm C}}g_{\rm T}^{(2)}(0)=0.074(21)\,\%,\tag{32}$$

$$\Delta S_{\rm impurity}\approx3\sqrt{2}\left(\frac{\eta_{\rm C}}{\eta_{\rm T}}g_{\rm C}^{(2)}(0)+\frac{\eta_{\rm T}}{\eta_{\rm C}}g_{\rm T}^{(2)}(0)\right)=0.0031(9),\tag{33}$$

where $g_{\rm C}^{(2)}(0)=g_{\rm T}^{(2)}(0)=2.9(8)\times10^{-4}$ is determined in the HBT experiment as shown in Section I, and $\eta_{\rm T}/\eta_{\rm C}=2.11(4)$ is given in Section III.

Effects from photon distinguishability. We characterize the photon indistinguishability using the same method as in Section III. By tracing the impact of distinguishable input mode $\hat{T}'_{\rm A}=-\hat{T}'_1$ on the correlations

$$\begin{aligned}
p(\alpha,\beta)&\approx\langle\hat{c}_\alpha^\dagger\hat{t}_\beta^\dagger\hat{t}_\beta\hat{c}_\alpha\rangle+\langle\hat{c}_\alpha^\dagger\hat{t}_\beta'^\dagger\hat{t}'_\beta\hat{c}_\alpha\rangle+\langle\hat{c}_\alpha'^\dagger\hat{t}_\beta^\dagger\hat{t}_\beta\hat{c}'_\alpha\rangle\\
&=\frac{1}{9}\langle\hat{C}_{\rm D}^\dagger\hat{C}_{\rm D}\rangle\left[\langle\hat{T}_{\rm A}^\dagger\hat{T}_{\rm A}\rangle\frac{\sin^2(\alpha-\beta)}{2}+\langle\hat{T}_{\rm A}'^\dagger\hat{T}'_{\rm A}\rangle\left(\sin^2\alpha\cos^2\left(\beta+\frac{\pi}{4}\right)+\cos^2\left(\alpha+\frac{\pi}{4}\right)\sin^2\beta\right)\right],
\end{aligned}\tag{34}$$

$$p({\rm L\backslash R},{\rm L\backslash R})\approx\langle\hat{c}_{\rm L\backslash R}^\dagger\hat{t}_{\rm L\backslash R}^\dagger\hat{t}_{\rm L\backslash R}\hat{c}_{\rm L\backslash R}\rangle+\langle\hat{c}_{\rm L\backslash R}^\dagger\hat{t}_{\rm L\backslash R}'^\dagger\hat{t}'_{\rm L\backslash R}\hat{c}_{\rm L\backslash R}\rangle+\langle\hat{c}_{\rm L\backslash R}'^\dagger\hat{t}_{\rm L\backslash R}^\dagger\hat{t}_{\rm L\backslash R}\hat{c}'_{\rm L\backslash R}\rangle=\frac{1}{18}\langle\hat{C}_{\rm D}^\dagger\hat{C}_{\rm D}\rangle\langle\hat{T}_{\rm A}'^\dagger\hat{T}'_{\rm A}\rangle,\tag{35}$$

$$\begin{aligned}
p({\rm L\backslash R},{\rm R\backslash L})&\approx\langle\hat{c}_{\rm L\backslash R}^\dagger\hat{t}_{\rm R\backslash L}^\dagger\hat{t}_{\rm R\backslash L}\hat{c}_{\rm L\backslash R}\rangle+\langle\hat{c}_{\rm L\backslash R}^\dagger\hat{t}_{\rm R\backslash L}'^\dagger\hat{t}'_{\rm R\backslash L}\hat{c}_{\rm L\backslash R}\rangle+\langle\hat{c}_{\rm L\backslash R}'^\dagger\hat{t}_{\rm R\backslash L}^\dagger\hat{t}_{\rm R\backslash L}\hat{c}'_{\rm L\backslash R}\rangle\\
&=\frac{1}{18}\langle\hat{C}_{\rm D}^\dagger\hat{C}_{\rm D}\rangle\Big(\langle\hat{T}_{\rm A}^\dagger\hat{T}_{\rm A}\rangle+\langle\hat{T}_{\rm A}'^\dagger\hat{T}'_{\rm A}\rangle\Big),
\end{aligned}\tag{36}$$

where $\hat{c}'$ and $\hat{t}'$ represent the distinguishable output modes, we derive

$$E(\theta_c,\theta_t) \approx \frac{\cos 2(\theta_c-\theta_t)\langle \hat{T}_A^\dagger \hat{T}_A\rangle - \sin 2(\theta_c+\theta_t)\langle \hat{T}_A'^\dagger \hat{T}_A'\rangle}{\langle \hat{T}_A^\dagger \hat{T}_A\rangle + 2\langle \hat{T}_A'^\dagger \hat{T}_A'\rangle}, \tag{37}$$

$$1+\langle \sigma_c^x \sigma_t^x\rangle = 1 - E\left(\frac{\pi}{4},\frac{\pi}{4}\right) \approx 2\epsilon_d = 0.0012(16), \tag{38}$$

$$1+\langle \sigma_c^y \sigma_t^y\rangle \approx 1 - \frac{\langle \hat{T}_A^\dagger \hat{T}_A\rangle}{\langle \hat{T}_A^\dagger \hat{T}_A\rangle + 2\langle \hat{T}_A'^\dagger \hat{T}_A'\rangle} \approx 2\epsilon_d = 0.0012(16), \tag{39}$$

$$1+\langle \sigma_c^z \sigma_t^z\rangle = 1 - E(0,0) \approx 2\epsilon_d = 0.0012(16), \tag{40}$$

and furthermore the reduction of Bell parameter and the infidelity of Bell state are given by

$$\Delta F_d^{\Psi^-} \approx \frac{3}{2}\epsilon_d = 0.09(12)\,\%, \tag{41}$$

$$\Delta S_d \approx 4\sqrt{2}\epsilon_d = 0.0033(44), \tag{42}$$

where the distinguishability $\epsilon_d = 0.06(8)\,\%$ is determined in the HOM interference experiment as shown in Section II.

Effects from imperfect optical components. The imperfections of optical components in the state preparation and gate-operation stage are fully characterized in Supplementary Equation (18). The imperfect operation $\mathbf{U}_{\mathrm{CNOT}}$ transforms the perfectly defined input state $(|0\rangle_C - |1\rangle_C)|1\rangle_T/\sqrt{2}$ to a flawed output state

$$\frac{\mathbf{U}_{\mathrm{CNOT}}}{\sqrt{2}}\begin{pmatrix}0\\1\\0\\-1\end{pmatrix} \approx \frac{1}{\sqrt{2}}\begin{pmatrix} \frac{\epsilon_{H_0}}{4}-\frac{\epsilon_{H_2}}{4}-\frac{\epsilon_{V_0}}{\sqrt{2}}+\frac{\epsilon_T}{2} \\ 1-\frac{\epsilon_{H_0}}{4}-\frac{\epsilon_{H_1}}{2}-\frac{\epsilon_{H_2}}{4}+\frac{\epsilon_{V_0}}{\sqrt{2}}-\frac{\epsilon_C}{2} \\ -1-\frac{3\epsilon_{H_0}}{4}+\frac{\epsilon_{H_2}}{4}-\frac{\epsilon_{V_0}}{\sqrt{2}}-\frac{\epsilon_C}{2} \\ \frac{5\epsilon_{H_0}}{4}+\frac{\epsilon_{H_2}}{4}-\frac{\epsilon_{V_0}}{\sqrt{2}}-\frac{\epsilon_T}{2} \end{pmatrix}, \tag{43}$$

which can be rewritten as $|\Psi^-\rangle + \epsilon_+|\Phi^+\rangle + \epsilon_-|\Phi^-\rangle + \epsilon_\Psi|\psi^+\rangle$ in terms of Bell basis. For this output, again, we first calculate the correlations

$$p(\alpha,\beta) \approx \frac{1}{18}\left[\sin(\alpha-\beta) - \cos(\alpha-\beta)\epsilon_+ - \cos(\alpha+\beta)\epsilon_- - \sin(\alpha+\beta)\epsilon_\psi\right]^2, \tag{44}$$

$$p(\mathrm{L\backslash R},\mathrm{L\backslash R}) \approx \frac{1}{18}\left(\epsilon_-^2+\epsilon_\psi^2\right), \tag{45}$$

$$p(\mathrm{L\backslash R},\mathrm{R\backslash L}) \approx \frac{1}{18}(1+\epsilon_+^2), \tag{46}$$

where $\epsilon_+ \approx 3\epsilon_{H_0}/4 - \epsilon_{V_0}/\sqrt{2} = -0.0056(38)$, $\epsilon_- \approx -\epsilon_{H_0}/2 - \epsilon_{H_2}/4 + \epsilon_T/2 = 0.0000(31)$ and $\epsilon_\psi \approx -\epsilon_{H_0}/2 - \epsilon_{H_1}/4 - \epsilon_C/2 = 0.0000(31)$. Considering the second-order effect of polarization crosstalk, we have

$$\Delta F_{\mathrm{optics}}^{\Psi^-} \approx \epsilon_+^2 + \epsilon_-^2 + \epsilon_\psi^2 + \frac{3}{4}\left(\epsilon_{c_0}^2+\epsilon_{c_1}^2+\epsilon_{t_0}^2+\epsilon_{t_1}^2\right) \approx 0.003(4)\,\% \tag{47}$$

$$\Delta S_{\mathrm{optics}} \approx 2\sqrt{2}\left(2\epsilon_+^2 + \epsilon_-^2 + \epsilon_\psi^2 + \epsilon_{c_0}^2+\epsilon_{c_1}^2+\epsilon_{t_0}^2+\epsilon_{t_1}^2\right) = 0.00018(24). \tag{48}$$

In summary, background detection events, single-photon impurity, photon distinguishability and imperfect optical elements reduce the state fidelity F_{Ψ^-} (Bell parameter S) from its ideal value by 0.123(4) % (0.00422(16)), 0.074(21) % (0.0031(9)), 0.09(12) % (0.0033(44)) and 0.003(4) % (0.00018(24)), respectively. Considering the above-mentioned effects, we expect a value of 99.71(12) % (2.818(5)) for F_{Ψ^-} (S), which is in good agreement with the measured value of 99.69(4) % (2.823(12)).

V. SUPPLEMENTARY NOTE 5: POTENTIAL APPLICATIONS

We demonstrate a near-optimal Rydberg atom single-photon source and realize a two-photon quantum logic gate with a high fidelity. Our results provide potential applications in photonic quantum information processing and distributed matter-light quantum architectures, as schematically shown in Supplementary Figure 7.

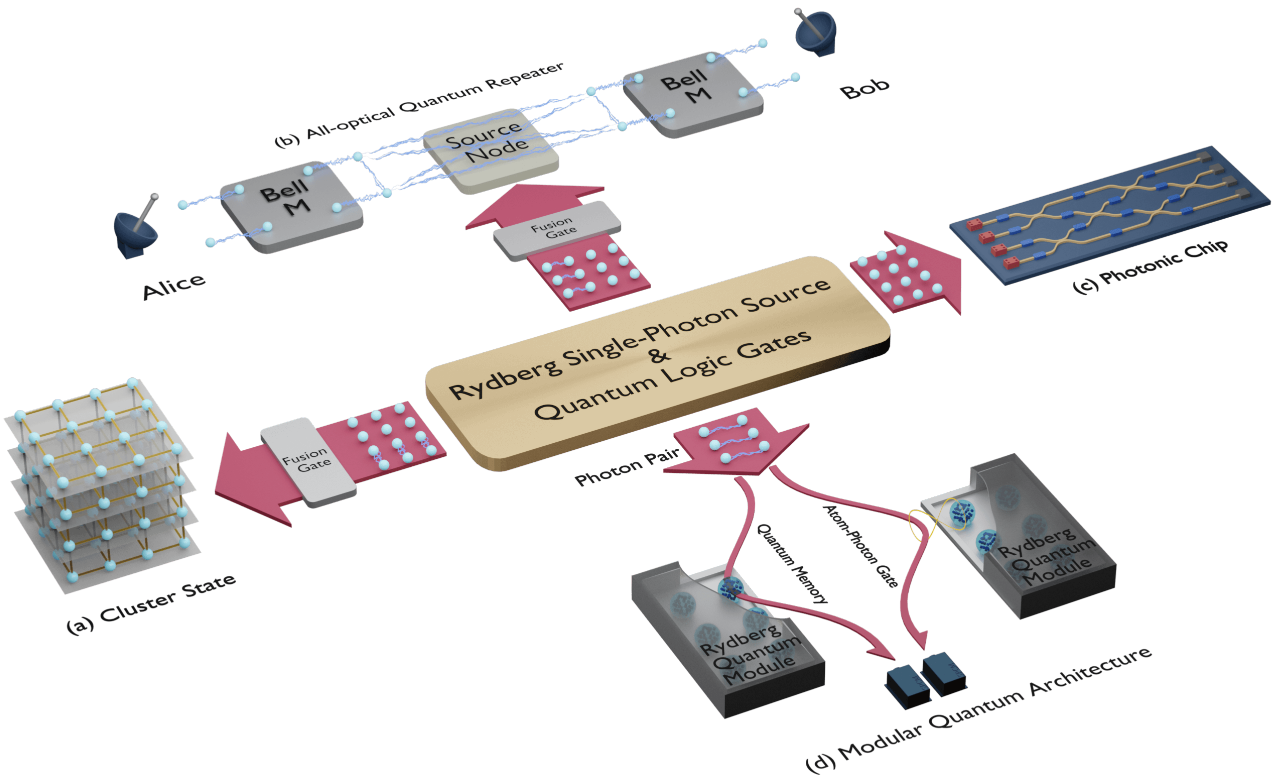

Supplementary Figure 7. Potential applications of Rydberg single-photon source and two-photon logic gate. **(a)** Build a three-dimensional cluster state that allows for fault-tolerant photonic quantum computing by using entangled photon pairs and single photons as resources. **(b)** Realize all-optical quantum repeaters by generating photonic cluster states using single photons and entangled photon pairs. **(c)** Inject single photons from Rydberg atoms into an integrated photonic chip to realize large-scale photonic quantum information processing. **(d)** Interconnect two Rydberg quantum modules using an entangled photon pair, with one of the single photons being stored in a module and the other being applied an atom-photon gate.

Measurement-based (or one-way) photonic quantum computing[2,67–69], which requires a large cluster-state as resource[70], is one of the most promising approaches to realize linear optical quantum computation. However, the main hindrance of the measurement-based photonic quantum computation is to efficiently generate high-fidelity cluster states. Our Rydberg single-photon source and demonstrated entangling gates provide a potential way to overcome this challenge. A collection of high-fidelity entangled Bell states, which are locally equivalent to two-qubit cluster states, can be used to generate a large cluster state by sequentially applying the fusion gates. Furthermore, the success probability of the fusion gate can be boosted by adding more ancillary single photons[71]. As an example, a three-dimensional cubic cluster state that allows for fault-tolerant photonic quantum computation[72,73] can be generated based on our system.

Achieving long-distance quantum communication[74,75] is very challenging. Due to the photon loss, the efficiency of quantum communication decreases exponentially with the communication distance. Quantum repeaters are thus introduced to beat the exponential scaling with the distance[76]. In the standard paradigm, a quantum repeater requires a quantum memory with a sufficiently long coherence time[76]. Alternatively, an all-optical quantum repeater circumvents the need for matter quantum memories by employing optical cluster states and single-qubit measurements[4]. Proof-of-principle experimental demonstrations of all-optical quantum repeaters have been realized using single photons prepared by spontaneous parametric down-conversion[5,77] and solid-state quantum emitters[78]. Our device can generate on-demand single photons and high-quality entangled photon pairs, which are building blocks for optical cluster states. Therefore, our Rydberg atom single-photon source and photon-photon gate play a central role in an all-optical quantum repeater and would be a key ingredient of the quantum internet.

A photonic chip provides a stable platform which can integrate a large number of reconfigurable optical components[54,60,62,79,80]. It is in principle possible to integrate all constituent parts that are required for photonic quantum information processing in a single chip[27,80–83], including single-photon sources, linear optical circuits and photon detec-

tors. However, it is challenging to maintain the performances of all constituent parts when combining them together. Given that Rydberg atoms can generate near-perfect single photons, it is natural to envision a new architecture which connects our Rydberg single-photon sources with a photonic chip, allowing for multi-photon quantum interference with high visibility. Near-future techniques will potentially enhance the single photon generation efficiency of Rydberg atoms to a level that enables large-scale quantum information processing on photonic chips.

The state-of-the-art quantum module consists of a relatively small number of matter qubits[84–86] (superconducting qubits, ion qubits, etc.) that can be precisely manipulated and mutually coupled via high-fidelity entangling gates. Increasing the number of qubits in a single quantum module would degrade the performance of the gate operations[87]. One potential solution is to connect many quantum modules using photons as intermediaries. Quantum correlations (logic gates) between quantum modules can be established (implemented) by either detecting photons emitted from two quantum modules[87], coupling a single photon sequentially with two quantum modules[88], or teleporting quantum gate using previously shared entanglement[89]. The overall performance of the quantum network relies critically on the quality of the single photons or entangled photon pairs. Our device therefore enables near-perfect interconnections between quantum modules and reduces the overall error rates of quantum networks[90].

Supplementary References

1 Knill, E., Laflamme, R. & Milburn, G. J. A scheme for efficient quantum computation with linear optics. *Nature* **409**, 46–52 (2001).

2 Browne, D. E. & Rudolph, T. Resource-efficient linear optical quantum computation. *Phys. Rev. Lett.* **95**, 010501 (2005).

3 O'Brien, J. L. Optical quantum computing. *Science* **318**, 1567–1570 (2007).

4 Azuma, K., Tamaki, K. & Lo, H.-K. All-photonic quantum repeaters. *Nat. Commun.* **6**, 6787 (2015).

5 Li, Z.-D. *et al.* Experimental quantum repeater without quantum memory. *Nat. Photon.* **13**, 644–648 (2019).

6 Hacker, B., Welte, S., Rempe, G. & Ritter, S. A photon-photon quantum gate based on a single atom in an optical resonator. *Nature* **536**, 193–196 (2016).

7 Tiarks, D., Schmidt-Eberle, S., Stolz, T., Rempe, G. & Dürr, S. A photon-photon quantum gate based on Rydberg interactions. *Nat. Phys.* **15**, 124–126 (2019).

8 Pittman, T. B., Jacobs, B. C. & Franson, J. D. Demonstration of nondeterministic quantum logic operations using linear optical elements. *Phys. Rev. Lett.* **88**, 257902 (2002).

9 Resch, K. J., Lundeen, J. S. & Steinberg, A. M. Conditional-phase switch at the single-photon level. *Phys. Rev. Lett.* **89**, 037904 (2002).

10 Franson, J. D., Jacobs, B. C. & Pittman, T. B. Experimental demonstration of quantum logic operations using linear optical elements. *Fortschr. Phys.* **51**, 369–378 (2003).

11 O'Brien, J. L., Pryde, G. J., White, A. G., Ralph, T. C. & Branning, D. Demonstration of an all-optical quantum controlled-NOT gate. *Nature* **426**, 264–267 (2003).

12 Langford, N. K. *et al.* Demonstration of a simple entangling optical gate and its use in Bell-state analysis. *Phys. Rev. Lett.* **95**, 210504 (2005).

13 Kiesel, N., Schmid, C., Weber, U., Ursin, R. & Weinfurter, H. Linear optics controlled-phase gate made simple. *Phys. Rev. Lett.* **95**, 210505 (2005).

14 Okamoto, R., Hofmann, H. F., Takeuchi, S. & Sasaki, K. Demonstration of an optical quantum controlled-NOT gate without path interference. *Phys. Rev. Lett.* **95**, 210506 (2005).

15 Crespi, A. *et al.* Integrated photonic quantum gates for polarization qubits. *Nat. Commun.* **2**, 566 (2011).

16 Pooley, M. A. *et al.* Controlled-NOT gate operating with single photons. *Appl. Phys. Lett.* **100**, 211103 (2012).

17 He, Y.-M. *et al.* On-demand semiconductor single-photon source with near-unity indistinguishability. *Nat. Nanotech.* **8**, 213–217 (2013).

18 Qiang, X. *et al.* Large-scale silicon quantum photonics implementing arbitrary two-qubit processing. *Nat. Photon.* **12**, 534–539 (2018).

19 Zhang, M. *et al.* Supercompact photonic quantum logic gate on a silicon chip. *Phys. Rev. Lett.* **126**, 130501 (2021).

20 Yuan, Z. *et al.* Electrically driven single-photon source. *Science* **295**, 102–105 (2002).

21 Ding, X. *et al.* On-demand single photons with high extraction efficiency and near-unity indistinguishability from a resonantly driven quantum dot in a micropillar. *Phys. Rev. Lett.* **116**, 020401 (2016).

22 Somaschi, N. *et al.* Near-optimal single-photon sources in the solid state. *Nat. Photon.* **10**, 340–345 (2016).

23 Schweickert, L. *et al.* On-demand generation of background-free single photons from a solid-state source. *Appl. Phys. Lett.* **112**, 093106 (2018).

24 Kwiat, P. G. *et al.* New high-intensity source of polarization-entangled photon pairs. *Phys. Rev. Lett.* **75**, 4337–4341 (1995).

25 Howell, J. C., Bennink, R. S., Bentley, S. J. & Boyd, R. W. Realization of the Einstein-Podolsky-Rosen paradox using momentum- and position-entangled photons from spontaneous parametric down conversion. *Phys. Rev. Lett.* **92**, 210403 (2004).

26 Zhong, H.-S. *et al.* 12-photon entanglement and scalable scattershot Boson sampling with optimal entangled-photon pairs from parametric down-conversion. *Phys. Rev. Lett.* **121**, 250505 (2018).

[27] Silverstone, J. W. *et al.* On-chip quantum interference between silicon photon-pair sources. *Nat. Photon.* **8**, 104–108 (2014).
[28] Yan, Z. *et al.* Generation of heralded single photons beyond 1100 nm by spontaneous four-wave mixing in a side-stressed femtosecond laser-written waveguide. *Appl. Phys. Lett.* **107**, 231106 (2015).
[29] Bernien, H. *et al.* Heralded entanglement between solid-state qubits separated by three metres. *Nature* **497**, 86–90 (2013).
[30] Shalaginov, M. Y. *et al.* Enhancement of single-photon emission from nitrogen-vacancy centers with TiN/(Al,Sc)N hyperbolic metamaterial. *Laser Photon. Rev.* **9**, 120–127 (2015).
[31] Dréau, A., Tchebotareva, A., El Mahdaoui, A., Bonato, C. & Hanson, R. Quantum frequency conversion of single photons from a nitrogen-vacancy center in diamond to telecommunication wavelengths. *Phys. Rev. Appl.* **9**, 064031 (2018).
[32] McKeever, J. *et al.* Deterministic generation of single photons from one atom trapped in a cavity. *Science* **303**, 1992–1994 (2004).
[33] Thompson, J. K., Simon, J., Loh, H. & Vuletić, V. A high-brightness source of narrowband, identical-photon pairs. *Science* **313**, 74–77 (2006).
[34] Dudin, Y. O. & Kuzmich, A. Strongly interacting Rydberg excitations of a cold atomic gas. *Science* **336**, 887–889 (2012).
[35] Peyronel, T. *et al.* Quantum nonlinear optics with single photons enabled by strongly interacting atoms. *Nature* **488**, 57–60 (2012).
[36] Mücke, M. *et al.* Generation of single photons from an atom-cavity system. *Phys. Rev. A* **87**, 063805 (2013).
[37] Maxwell, D. *et al.* Storage and control of optical photons using Rydberg polaritons. *Phys. Rev. Lett.* **110**, 103001 (2013).
[38] Baur, S., Tiarks, D., Rempe, G. & Dürr, S. Single-photon switch based on Rydberg blockade. *Phys. Rev. Lett.* **112**, 073901 (2014).
[39] Padrón-Brito, A., Lowinski, J., Farrera, P., Theophilo, K. & de Riedmatten, H. Probing the indistinguishability of single photons generated by Rydberg atomic ensembles. Preprint at https://arxiv.org/abs/2011.06901 (2020).
[40] Ripka, F., Kübler, H., Löw, R. & Pfau, T. A room-temperature single-photon source based on strongly interacting Rydberg atoms. *Science* **362**, 446–449 (2018).
[41] Petrosyan, D. & Mølmer, K. Deterministic free-space source of single photons using Rydberg atoms. *Phys. Rev. Lett.* **121**, 123605 (2018).
[42] Li, J. *et al.* Semideterministic entanglement between a single photon and an atomic ensemble. *Phys. Rev. Lett.* **123**, 140504 (2019).
[43] Ornelas-Huerta, D. P. *et al.* On-demand indistinguishable single photons from an efficient and pure source based on a Rydberg ensemble. *Optica* **7**, 813–819 (2020).
[44] Keller, M., Lange, B., Hayasaka, K., Lange, W. & Walther, H. Continuous generation of single photons with controlled waveform in an ion-trap cavity system. *Nature* **431**, 1075–1078 (2004).
[45] Maunz, P. *et al.* Quantum interference of photon pairs from two remote trapped atomic ions. *Nat. Phys.* **3**, 538–541 (2007).
[46] Crocker, C. *et al.* High purity single photons entangled with an atomic qubit. *Opt. Express* **27**, 28143–28149 (2019).
[47] Meraner, M. *et al.* Indistinguishable photons from a trapped-ion quantum network node. *Phys. Rev. A* **102**, 052614 (2020).
[48] Pittman, T., Fitch, M., Jacobs, B. & Franson, J. Experimental controlled-not logic gate for single photons in the coincidence basis. *Phys. Rev. A* **68**, 032316 (2003).
[49] O'Brien, J. L. *et al.* Quantum process tomography of a controlled-not gate. *Phys. Rev. Lett.* **93**, 080502 (2004).
[50] Huang, Y.-F., Ren, X.-F., Zhang, Y.-S., Duan, L.-M. & Guo, G.-C. Experimental teleportation of a quantum controlled-not gate. *Phys. Rev. Lett.* **93**, 240501 (2004).
[51] Gasparoni, S., Pan, J.-W., Walther, P., Rudolph, T. & Zeilinger, A. Realization of a photonic controlled-not gate sufficient for quantum computation. *Phys. Rev. Lett.* **93**, 020504 (2004).
[52] Zhao, Z. *et al.* Experimental demonstration of a nondestructive controlled-not quantum gate for two independent photon qubits. *Phys. Rev. Lett.* **94**, 030501 (2005).
[53] Bao, X.-H. *et al.* Optical nondestructive controlled-not gate without using entangled photons. *Phys. Rev. Lett.* **98**, 170502 (2007).
[54] Politi, A., Cryan, M. J., Rarity, J. G., Yu, S. & O'Brien, J. L. Silica-on-silicon waveguide quantum circuits. *Science* **320**, 646–649 (2008).
[55] Chen, J. *et al.* Demonstration of a quantum controlled-not gate in the telecommunications band. *Phys. Rev. Lett.* **100**, 133603 (2008).
[56] Patel, M., Altepeter, J. B., Hall, M. A., Medic, M. & Kumar, P. Experimental characterization of a telecommunications-band quantum controlled-not gate. *IEEE J. Sel. Top. Quantum Electron.* **15**, 1685–1693 (2009).
[57] Gao, W.-B. *et al.* Teleportation-based realization of an optical quantum two-qubit entangling gate. *Proc. Natl Acad. Sci. USA* **107**, 20869–20874 (2010).
[58] Lemr, K. *et al.* Experimental implementation of the optimal linear-optical controlled phase gate. *Phys. Rev. Lett.* **106**, 013602 (2011).
[59] Okamoto, R., O'Brien, J. L., Hofmann, H. F. & Takeuchi, S. Realization of a knill-laflamme-milburn controlled-not photonic quantum circuit combining effective optical nonlinearities. *Proc. Natl Acad. Sci. USA* **108**, 10067–10071 (2011).
[60] Shadbolt, P. J. *et al.* Generating, manipulating and measuring entanglement and mixture with a reconfigurable photonic circuit. *Nat. Photon.* **6**, 45–49 (2012).
[61] Gazzano, O. *et al.* Entangling quantum-logic gate operated with an ultrabright semiconductor single-photon source. *Phys. Rev. Lett.* **110**, 250501 (2013).
[62] Carolan, J. *et al.* Universal linear optics. *Science* **349**, 711–716 (2015).
[63] Zeuner, J. *et al.* Integrated-optics heralded controlled-not gate for polarization-encoded qubits. *npj Quantum Inf.* **4**, 1–7 (2018).

[64] Zhang, Q. *et al.* Femtosecond laser direct writing of an integrated path-encoded cnot quantum gate. *Opt. Mater. Express* **9**, 2318–2326 (2019).
[65] Li, J.-P. *et al.* Heralded nondestructive quantum entangling gate with single-photon sources. *Phys. Rev. Lett.* **126**, 140501 (2021).
[66] Feng, L.-T. *et al.* Transverse mode-encoded quantum gate on a silicon photonic chip. *Phys. Rev. Lett.* **128**, 060501 (2022).
[67] Nielsen, M. A. Optical quantum computation using cluster states. *Phys. Rev. Lett.* **93**, 040503 (2004).
[68] Bombin, H. *et al.* Interleaving: modular architectures for fault-tolerant photonic quantum computing. Preprint at https://arxiv.org/abs/2103.08612 (2021).
[69] Bartolucci, S. *et al.* Fusion-based quantum computation. Preprint at https://arxiv.org/abs/2101.09310 (2021).
[70] Raussendorf, R., Browne, D. E. & Briegel, H. J. Measurement-based quantum computation on cluster states. *Phys. Rev. A* **68**, 022312 (2003).
[71] Ewert, F. & van Loock, P. 3/4-efficient Bell measurement with passive linear optics and unentangled ancillae. *Phys. Rev. Lett.* **113**, 140403 (2014).
[72] Raussendorf, R. & Harrington, J. Fault-tolerant quantum computation with high threshold in two dimensions. *Phys. Rev. Lett.* **98**, 190504 (2007).
[73] Raussendorf, R., Harrington, J. & Goyal, K. Topological fault-tolerance in cluster state quantum computation. *New J. Phys.* **9**, 199 (2007).
[74] Lo, H.-K., Curty, M. & Tamaki, K. Secure quantum key distribution. *Nat. Photon.* **8**, 595–604 (2014).
[75] Pan, J.-W. *et al.* Multiphoton entanglement and interferometry. *Rev. Mod. Phys.* **84**, 777–838 (2012).
[76] Sangouard, N., Simon, C., de Riedmatten, H. & Gisin, N. Quantum repeaters based on atomic ensembles and linear optics. *Rev. Mod. Phys.* **83**, 33–80 (2011).
[77] Hasegawa, Y. *et al.* Experimental time-reversed adaptive Bell measurement towards all-photonic quantum repeaters. *Nat. Commun.* **10**, 378 (2019).
[78] Buterakos, D., Barnes, E. & Economou, S. E. Deterministic generation of all-photonic quantum repeaters from solid-state emitters. *Phys. Rev. X* **7**, 041023 (2017).
[79] Wang, J. *et al.* Multidimensional quantum entanglement with large-scale integrated optics. *Science* **360**, 285–291 (2018).
[80] Wang, J., Sciarrino, F., Laing, A. & Thompson, M. G. Integrated photonic quantum technologies. *Nat. Photon.* **14**, 273–284 (2020).
[81] Gerrits, T. *et al.* On-chip, photon-number-resolving, telecommunication-band detectors for scalable photonic information processing. *Phys. Rev. A* **84**, 060301 (2011).
[82] Pernice, W. H. P. *et al.* High-speed and high-efficiency travelling wave single-photon detectors embedded in nanophotonic circuits. *Nat. Commun.* **3**, 1325 (2012).
[83] Arcari, M. *et al.* Near-unity coupling efficiency of a quantum emitter to a photonic crystal waveguide. *Phys. Rev. Lett.* **113**, 093603 (2014).
[84] Debnath, S. *et al.* Demonstration of a small programmable quantum computer with atomic qubits. *Nature* **536**, 63–66 (2016).
[85] Zhang, J. *et al.* Observation of a many-body dynamical phase transition with a 53-qubit quantum simulator. *Nature* **551**, 601–604 (2017).
[86] Arute, F. *et al.* Quantum supremacy using a programmable superconducting processor. *Nature* **574**, 505–510 (2019).
[87] Monroe, C. *et al.* Large-scale modular quantum-computer architecture with atomic memory and photonic interconnects. *Phys. Rev. A* **89**, 022317 (2014).
[88] Daiss, S. *et al.* A quantum-logic gate between distant quantum-network modules. *Science* **371**, 614–617 (2021).
[89] Wan, Y. *et al.* Quantum gate teleportation between separated qubits in a trapped-ion processor. *Science* **364**, 875–878 (2019).
[90] Kimble, H. J. The quantum internet. *Nature* **453**, 1023–1030 (2008).
